# Mechanism of ferroptosis in a rat model of premature ovarian insufficiency induced by cisplatin

**DOI:** 10.1038/s41598-023-31712-7

**Published:** 2023-03-17

**Authors:** Rong Du, Xi Cheng, Jingjing Ji, Yang Lu, Yuanyuan Xie, Weina Wang, Yanhua Xu, Yuquan Zhang

**Affiliations:** 1 Department of Obstetrics and Gynecology, Affiliated Hospital of Nantong University, Medical School of Nantong University, Nantong, Jiangsu Province, 226001 China; 2 Department of Orthopedics, Affiliated Hospital of Nantong University, Nantong, Jiangsu Province, 226001 China; 3 Department of Obstetrics and Gynecology, Affiliated Hospital of Nantong University, No.20, Xisi Road, Nantong, Jiangsu Province, 226001 China

**Keywords:** Biochemistry, Cell biology, Diseases, Endocrinology, Medical research, Molecular medicine, Pathogenesis

## Abstract

Ferroptosis is widely present in fibrosis-related diseases. The basic pathology of premature ovarian insufficiency (POI) involves ovarian tissue fibrosis, and there are currently fewer relevant studies addressing the association between ferroptosis and POI. This study aimed to demonstrate that ferroptosis induced by cisplatin (CDDP) caused ovarian tissue fibrosis, leading to POI. Vitamin E (VE), a ferroptosis inhibitor, could repair damaged ovarian function. CDDP was used to establish a rat model of POI, and VE was administered to reverse the reproductive toxicity of CDDP. Ovarian function was assessed by histological section staining, follicle counts, sex hormone levels, as well as fertility assays. The extent of ferroptosis was assessed by transmission electron microscopy (TEM), malondialdehyde (MDA), Perls staining. CCK-8, Ethynyl-2-Deoxyuridine (EdU), and scratch assays were used to determine the effect of CDDP and VE on ovarian granulosa cell (GC) viability. Western blot, quantitative reverse-transcription polymerase chain reaction (qRT-PCR) and immunohistochemistry were performed to evaluate ferroptosis-related molecular changes. Our results showed that CDDP caused follicle development disorders and ovarian tissue fibrosis, the levels of sex hormones suggested impaired ovarian function, and VE could reverse the reproductive toxicity of CDDP. The results of TEM, MDA and Perls staining suggested that the typical mitochondrial signature of ferroptosis was altered in ovarian GCs from the CDDP group, with significantly higher levels of lipid peroxidation and significant iron deposition in ovarian tissue, whereas VE mitigated the extent of ferroptosis. Molecular experiments then confirmed that the ferroptosis-related molecules acetyl CoA synthetase long chain family member 4 (ACSl4), 15-lipoxygenase-1 (ALOX15), solute carrier family 7 member 11 (SLC7A11), and glutathione peroxidase 4 (GPX4) were differentially expressed in each group. In summary, our study preliminarily demonstrated that CDDP may promote GCs to undergo ferroptosis, cause follicle development disorders, ovarian tissue fibrosis, and induce POI by regulating the expression of ACSl4, ALOX15, SLC7A11, and GPX4, while VE improved impaired ovarian function.

## Introduction

Guidelines issued by the European Society of Human Reproduction and Embryology (ESHRE) in 2016 state that the diagnostic criteria for premature ovarian insufficiency (POI) are as follows: loss of ovarian activity before the age of 40 years, the appearance of oligomenorrhea or amenorrhea for ≥ 4 months, and at least two episodes of basal follicle-stimulating hormone (FSH) > 25 mIU/mL (measured > 4 weeks apart)^1^. In young cancer patients, iatrogenic factors, such as chemotherapy, have toxic effects on the reproductive system of young women and are one of the important etiologies predisposing to POI^2^. Previous studies have suggested that the main pathogenic mechanism of POI caused by chemotherapy may involve ovarian hypofunction by inducing oocyte apoptosis or disrupting granulosa cells (GCs) function, causing depletion of primordial follicles, an increase in atretic follicles, and fibrosis of ovarian tissue^3,4^. As conventional treatments such as hormone replacement are not effective in improving ovarian function and improving fertility^5,6^, there is a great need to confirm the existence of other unknown pathogenic mechanisms as well as other effective treatments.

Ferroptosis, first proposed in 2012 by Dixon et al., is an iron-mediated novel programmed cell death, and the main mechanism involves the action of divalent iron or lipoxygenases, which catalyze unsaturated fatty acids highly expressed on the cell membrane, and undergo lipid peroxidation, thereby inducing cell death^7–9^. The main morphological features that distinguish ferroptosis from apoptosis, autophagy, or necrosis are the presence of normal sized nuclei without nuclear condensation, but ultrastructurally, they show smaller mitochondria with reduced or absent mitochondrial cristae^10^.

An increasing number of studies have identified iron accumulation and lipid peroxide accumulation in the pathology of many fibrotic diseases, including liver fibrosis, pulmonary fibrosis, myocardial fibrosis, and renal fibrosis, and the use of ferroptosis inhibitors can reverse the progression of organ fibrosis, suggesting that ferroptosis is involved in the development of fibrotic diseases^11–17^. It is well-documented that the basic pathological changes leading to POI by cisplatin (CDDP) were mainly ovarian tissue fibrosis and follicle development disorders, which were prevented by the use of fibrosis-associated molecules (transforming growth factor-β1) inhibitors, which reversed fibrosis progression and improve ovarian function^18^. CDDP has also been shown to act as a ferroptosis inducer targeting glutathione (GSH), promoting cell ferroptosis^19,20^. Therefore, we propose the hypothesis that the mechanism of action of CDDP causing POI may be related to ferroptosis.

In this study, we used an integrated biological approach to investigate whether ferroptosis mediated the formation of POI caused by CDDP and to investigate the underlying mechanism. This is among the few reports reporting an association between ferroptosis and POI. We propose the following hypotheses: 1. CDDP causes lipid peroxidation in the GC membrane of the ovary and promotes cell ferroptosis, thereby causing follicle development disorders, ovarian tissue fibrosis, and finally affecting ovarian function, leading to fertility loss; 2. Ferroptosis is associated with POI with changes in ferroptosis-related molecule expression in POI, and the use of the ferroptosis inhibitor Vitamin E can inhibit ovarian GCs from undergoing ferroptosis, resulting in improved ovarian function. Our validation of these hypotheses provided experimental evidence and new insights into the pathophysiology of chemotherapy-induced POI.

## Materials and methods

### Animal ethics

SPF grade 3 or 4-weeks-old and 7-weeks-old female SD rats, and 8-weeks-old male SD rats were provided by the Laboratory Animal Center of Nantong University in Jiangsu Province using the license number SYXK (Su) 2017–0046. During the experimental period, rats were housed in a barrier environment with a 12:12 h light–dark cycle SPF grade, with free access to food and water at a room temperature of 23 ± 3 °C, and the litters was clean, dry and replaced regularly. Laboratory Animal Center of Nantong University in Jiangsu Province approved the protocols for animal experiments (No: S20221028-001), and all methods were performed in accordance with ARRIVE guidelines and relevant and regulations.

### Animal model

Forty-eight 7-weeks-old SD female rats were randomly assigned to four groups (n = 12/group): Control, POI (CDDP), phosphate-buffered saline (PBS) treated (CDDP + PBS), and Vitamin E treated (CDDP + VE). In the POI group, CDDP (2.0 mg/kg/d) was injected intraperitoneally (Qilu Pharmaceutical Co., Ltd., Shandong, China) for 7 consecutive days; the PBS treated group was then gavaged with PBS 0.2 mL at the same time as the CDDP injection, and after CDDP administration, PBS gavage was continued until the samples were taken; the Vitamin E-treated group was gavaged with VE 100 mg/kg (Sigma-Aldrich, Shanghai, China) at the same time as CDDP injection, and after CDDP administration, VE gavage was continued until the samples were taken. The control group received PBS 1 mL intraperitoneally for 7 consecutive days. After induction of anesthesia with isoflurane, six rats from each group were randomly selected on the 17th day of the experiment, and serum and ovaries were rapidly dissected and collected.

### Estrous cycle determination

Before the experiment, the estrous cycle was determined in the rats, and the animals with a normal estrous cycle were selected for the experiment. The normal estrous cycle in rats is usually 4 − 5 days and consists of four regular and continuous phases: P (proestrus), E (estrus), M (metestrus) and D (diestrus). For 17 days, vaginal smears were taken daily (8 am–10 am) using normal saline, pipette tips and pipettes. The smears were dried naturally in air and then subjected to modified Giemsa staining (Beyotime, Beijing, China), and observed under the microscope. Microscopically, Vaginal cells were mainly leukocytes during diestrus, nucleated cells were the main type during proestrus, and fragmented keratinized epithelial cells were the main type during estrus, while leukocytes, nucleated cells, and epithelial cells were all found in the late metestrus stage^21^.

### Serum sex hormone assays

After 2 h at room temperature, the collected rat serum was centrifuged at 3000 × *g* for 15 min to obtain the supernatant and stored at − 80 °C for analysis. Serum levels of estradiol (E2), follicle-stimulating hormone (FSH) and anti-Mullerian hormone (AMH) were measured using ELISA kits following the manufacturer's instructions (Elabscience, Wuhan, China). Absorbance was detected with a multifunctional microplate reader (BioTek, USA), and the standard curve was calculated and concentration conversions were performed using a 4-parameter fitting logistic function.

### HE staining and follicle counting

Ovaries that had been immersed overnight in 4% paraformaldehyde solution were removed and placed in embedding boxes, which were routinely dehydrated and embedded in paraffin. Tissue wax blocks were serially sectioned (5 μm), and then every fifth section was stained with hematoxylin/eosin (HE) for the nucleus/cytoplasm. The stained sections were observed under a light microscope. To avoid repeated counting of the same follicle, only follicles with visible oocyte nuclei were counted. To analyze the ovarian morphology and count the numbers of ovarian follicles, the slides were examined under a light microscope. The follicles were categorized as primordial follicles, primary follicles, secondary follicles, mature follicles and atretic follicles^22^. Primordial follicles: located in the superficial layers of the ovarian cortex, were small in size and numerous, and consisted of an intermediate primary oocyte and a surrounding single layer of flattened GCs; primary follicles: composed of a primary oocyte and a monolayer of cuboidal GCs coating the oocyte and are scored as a primary follicle when the GCs had both flat and cuboidal morphologies with a cuboidal predominance; secondary follicles: an oocyte with a visible nucleolus and more than one layer of granulosa or thecal cells; mature follicles were the largest of all grades of follicles, with an increase in follicular fluid extruding oocytes and the surrounding GCs to one side; atretic follicles: irregular oocyte morphology, pyknotic or dissolved nuclei, concave follicle walls, and collapse of the zona pellucida. Granulosa cells and theca cells were loosely arranged and sloughed off with cell atrophy into the follicular cavity. All HE follicle counting fractions were examined by two observers.

### Masson trichrome staining

Ovaries were collected from the rats in each group and fixed in 4% paraformaldehyde for 24 h. Following paraffin embedding and sectioning (5 μm), the tissues were stained with Masson's Trichrome Stain Kit (Servicebio, Wuhan, China). To evaluate fibrosis of the ovarian tissues in each group, the slides were analyzed and photographed under a light microscope. Five fields in each staining image were randomly selected for examination. ImageJ v1.4 (National Institutes of Health, Bethesda, MD, USA) software was used to quantitate the degree of fibrosis in the ovarian tissue.

### Immunohistochemistry

Ovarian tissue paraffin Sects. (4 µm) were subjected to manual immunohistochemical staining. The steps were as follows: a. oven at 60 °C for 2 h, rehydration in graded alcohol after deparaffinization in dimethylbenzene; b. incubate with 3% H_2_O_2_ at room temperature for 10 min, wash with distilled water three times for 3 min each time; c. citrate antigen retrieval hydrothermal repair; d. 5% BSA blocking for 30 min at room temperature; e. primary antibodies against ACSl4 (1:50 Santa Cruz Biotechnology, USA), ALOX15 (1:4,000 Abcam, UK), SLC7A11 (1:200 Proteintech, Wuhan, China), and GPX4 (1:50 Abmart, Shanghai, China) were incubated overnight at 4 °C; f. incubated with biotinylated secondary antibodies (Boster Biological Technology Co., Ltd., Wuhan, China) at 37 °C for 30 min; g. staining was accomplished microscopically using a DAB Kit (Boster Biological Technology). Microscopic images were obtained, and the brownish yellow area in ovarian tissue was quantitatively assessed with ImageJ software. As a control for the immunostaining procedure, some sections were incubated with PBS instead of the primary antibody. No false positive reactions were detected in the sections.

### Perls staining

After referring to the Prussian Blue Iron Stain Kit (with eosin) (Solarbio, Beijing, China) protocol, paraffin sections of ovarian tissues (5 μm) were baked at 60 °C for 1 h, deparaffinized in dimethylbenzene and graded alcohol to water. The sections were further immersed in staining reagent Perls stain A for 10 min, then rinsed thoroughly in distilled water and immersed in eosin staining solution for 30 s. Slides were finally dehydrated in graded alcohol, cleared in dimethylbenzene, and mounted in neutral resin for microscopic examination.

### Transmission electron microscopy (TEM)

Ovarian tissues were rapidly cut into sections approximately 1 mm^3^ in size using a sharp blade, and fixed with 4% paraformaldehyde glutaraldehyde mixture fixative (Leagene Biotechnology, Beijing, China) for 2 h at room temperature in the dark, then transferred to 4 °C for storage, followed by dehydration, drying, and embedding. Finally, ultrathin sections were prepared to observe the samples under a transmission electron microscope (Hitachi, Tokyo, Japan).

#### Malondialdehyde (MDA) assay

Ovarian tissue sections were rinsed in ice cold normal saline to remove blood, and dried with filter paper, accurately weighed, and placed into 2.5 mL homogenate tubes. Nine volumes of 0.86% saline were added to the homogenate tubes at a ratio of weight (g): volume (mL) = 1:9, and the tissue homogenates were prepared using a tissue homogenizer (Shanghai Jing Xin, Shanghai, China) at 4 °C under the following homogenization conditions: 12,000 − 15,000 rpm for 10 s/time with 30 s gap for 3 − 5 consecutive times. The prepared 10% homogenates were centrifuged at 2500 × *g* for 10–15 min at 4 °C in a low speed centrifuge (Eppendorf centrifuge 5417R, Germany), and the supernatants were collected. The loading of reagents and samples was performed according to the malondialdehyde (MDA) assay kit (TBA method) (Nanjing Jiancheng Bioengineering Institute, Nanjing, China) instructions, and the reaction was monitored at 532 nm by a microplate reader (BioTek).

#### Fertility examination

Six rats were randomly selected from each group. Female rats were mated with sexually mature male rats at a 2:1 ratio. The vaginal plug was observed every morning at 08:00 am to determine if mating was successful. Following detection of the vaginal plug, the rats were euthanized at 17 − 18 days of gestation, the number of pregnancies and litter size were observed and examined for litter appearance. The observation time was calculated from the beginning of caging until 6 months after caging.

#### Cell extraction, culture and identification

The 3 or 4-weeks-old rats were injected intraperitoneally with pregnant mare serum gonadotropin (PMSG) (Ningbo Second Hormone Factory, Ningbo, Zhejiang, China) at a dose of 80 IU. The rats were then anesthetized with isoflurane 48 h later, the bilateral ovaries were removed under sterile conditions, and the antral follicles were punctured using a sterile syringe (Olympus, Japan) under a stereoscope. The remaining ovarian tissue was cut into tissue blocks approximately 1 mm^3^ in size with ophthalmic scissors and digested in 0.25% Trypsin–EDTA (Gibco, USA) for 5 − 10 min. The tissues were then resuspended in serum-free DMEM/nutrient mixture F-12 (Sigma-Aldrich, USA), and the oocytes as well as surrounding GCs were simultaneously extruded by gentle rubbing pressure and homogenized by repeated pipetting before being placed in a 70 μm nylon mesh (BD Falcon, USA) and filtered to separate GCs from oocytes. The filtrate was centrifuged at 1000 × *g* for 5 min using a tabletop low speed centrifuge (Hexiyiqi, Hunan, China), and the supernatant was discarded. The centrifuged cell pellet was cultured in complete medium, including DMEM / nutrient mixture F-12 medium supplemented with 10% fetal bovine serum (Gibco, USA), 1% 100 IU/mL penicillin and 100 IU mL streptomycin, and then placed in a 37 °C humidified incubator with 5% CO_2_. Cells at passage P3 were used for subsequent experiments.

GCs were identified by immunofluorescence cytochemical staining and the FSH receptor (FSHR), a specific marker of ovarian GCs was observed. Cells adherent to small round pieces were fixed using 4% paraformaldehyde for 30 min. After PBS washing, the cells were blocked using goat serum for 30 min at room temperature. Primary anti-FSHR rabbit polyclonal antibody (1:50, Proteintech) was added dropwise overnight at 4 °C. Goat anti-rabbit IgG and Alexa Fluor 488 (1:1,000, Abcam) were added dropwise the next day and incubated for 2 h in the dark. Antifade Mounting Medium with Hoechst 33,342 (Beyotime) was added dropwise onto the slides. The staining of cells was observed using a fluorescence inverted microscope (Olympus, Tokyo, Japan).

#### Cell model (CCK8 cell viability assay)

The effects of different concentrations of CDDP and the ferroptosis inhibitor Vitamin E on GCs viability were measured using a CCK-8 Kit (Dojindo, Japan). GCs (10,000/well) were seeded in 96 well plates and incubated overnight. When the cells had grown to 80 − 90% confluence, the medium was changed to one containing CDDP (0, 1, 3, 5, 8, and 10 μg/mL) or CDDP (5 μg/mL) + VE (0, 50, 100, 200, 400 µmol/L). After 24 h, 10 μL CCK-8 was added to each well and the absorbance at 450 nm was determined using a microplate reader (BioTek) at 1, 2, and 4 h after the addition of CCK-8. A model to determine the effects of CDDP and VE on the viability of GCs was established. Each experiment was repeated three times with five replicate wells per group.

#### Ethynyl-2-deoxyuridine (EdU) assay

The EdU proliferation assay was carried out using the BeyoClick™ EdU Cell Proliferation Kit with Alexa Fluor 488 (Beyotime). Briefly, GCs in the logarithmic growth phase were seeded in 24 well plates (50,000 cells/well) containing small discs and then the cells were divided into the following three groups: Control, CDDP, and CDDP + VE. After 24 h of culture, the control medium was replaced with complete medium, and the CDDP medium was replaced with medium containing CDDP (5 μg/mL) in complete medium, and the medium from the CDDP + VE group was replaced with medium containing CDDP (5 μg/mL) and VE (200 μmol/L) in complete medium. After 24 h stimulation, the EdU reaction solution was added to each well and incubated for a further 2 h according to the manufacturer’s instructions. The medium was removed when EdU incubation was complete, and the cells were washed with PBS before fixing with 4% paraformaldehyde for 30 min. Permeabilization, washing, and addition of reaction solution were performed according to the manufacturer’s instructions. After incubation for 30 min in the dark at room temperature, the cells were washed with washing solution three times. Antifade Mounting Medium with Hoechst 33,342 (Beyotime) was added dropwise onto the slides, which were then observed and photographed using a fluorescence inverted microscope (Olympus). The number of EdU positive cells was calculated with ImageJ software, and cell proliferation was calculated by dividing the number of EdU positive cells by the total number of cells.

#### Wound healing assay

On the 6-well plate, straight transverse lines were drawn evenly through the holes at 0.5 ~ 1 cm apart with a marker pen, passing at least five lines per well. Cells were seeded in each well with 1 × 10^6^ GCs and after 24 h of culture, when confluence was approximately 100%, a scratch was made with a pipette tip as perpendicular as possible to the transverse line. Grouping was the same as that for the EdU experiment, and the cells were divided into the following three groups: Control, CDDP, and CDDP + VE. The cells were washed three times using sterile PBS to remove the scratched cells, the control medium was changed to serum-free medium, and the CDDP medium was changed to medium containing CDDP (5 μg/mL) in serum-free medium, and the medium in the CDDP + VE group was replaced with medium containing CDDP (5 μg/mL) and VE (200 μmol/L) in serum-free medium. Photographs were obtained after the cells were placed in the incubator for continued culture for 0, 6, 12, and 24 h. The clean area was calculated by ImageJ software. The relative clean area was calculated by dividing the clean area measured at 6, 12 and 24 h by the clean area measured in 0 h.

#### Western blotting

Ovarian tissues and GCs were lysed using the Tissue or Cell Total Protein Extraction Kit (Sangon Biotech, Shanghai, China), and protein concentrations were detected using a BCA kit (Thermo, USA). Equal amounts of protein were dissolved in 5 × loading buffer, and electrophoresed on 7.5% or 10% SDS polyacrylamide gels and transferred to PVDF membranes. After blocking with 5% skim milk, the membranes were incubated with anti-FACL4 (1:10,000, ab155282, Abcam); anti-15Lipoxygenase antibody (1:1000, ab244205, Abcam); anti-xCT (1:1000, ab175186, Abcam); anti-GPX4 antibody (1:1000, TD6701, Abmart); anti-β-actin (1:20,000, Sigma-Aldrich) primary antibodies overnight. After incubation, the membranes were washed with TBS and Tween 20 (TBST) three times and then immunoblotted with HRP-conjugated secondary antibodies (1:2000, Proteintech) for 2 h at room temperature. The expression of each protein was measured by an enhanced chemiluminescence reagent (ECL) kit (Beyotime). The density of the protein expression bands was measured using ImageJ software.

#### Quantitative reverse-transcription polymerase chain reaction (qRT-PCR)

Total RNA was isolated from ovarian tissues and GCs using Trizol reagent (Ambion, USA), and RNA was reverse transcribed into cDNA using HiScript ii Q RT supermix for qPCR (+ gDNA wiper) (Vazyme, Nanjing, China). ChamQ universal SYBR qPCR Master Mix (Vazyme) was added to the PCR reaction system, and then loaded into an octaplex tube (Kirgen, Shanghai, China) as follows (predenaturation 95 °C for 30 s; cycling reaction: 95 °C for 10 s, 60 °C for 30 s, 40 cycles; dissolution curve 95 °C for 15 s, 60 °C for 60 s, 95 °C for 15 s) in a thermal cycler (Robocycler Gradient 96, BIOMETRA®, Princeton, NJ, USA) to run the reaction. The primers for qRT-PCR were as follows: ACSL4 forward primer: CCATATCGCTCTGTCACGCACTTC and reverse primer: CCAGGCTGTCCTTCTTCCCAAAC; ALOX15 forward primer: AGCTGTGCAAGACGACTATGAACTG and reverse primer: CGGGACTGAAGAGAGGTAGGGAAG; SLC7A11 forward primer: CCATCATCATCGGCACCGTCATC and reverse primer: TACTCCACAGGCAGACCAGAACAC; GPX4 forward primer: ATAAGAACGGCTGCGTGGTGAAG and reverse primer: TAGAGATAGCACGGCAGGTCCTTC; 18S forward primer: AGTCCCTGCCCTTTGTACACA and reverse primer: CGTTCCGAGGGCCTCACT. The housekeeping gene 18S was used to normalize gene expression, and tests for each group were repeated in triplicate. Results were analyzed using the Livak method (= 2^−ΔΔCT^) to estimate the fold change for each gene.

#### Data analysis

When the data showed normal distribution and homogeneity of variance, the student's t-test (two groups) or one-way analysis of variance (multiple groups) was used for data analysis. Otherwise, the Mann–Whitney U test (two groups) or Kruskal Wallis test (multiple groups) was employed. The LSD method was used after one-way ANOVA as the post hoc test and the Bonferroni method after performing the Kruskal Wallis test. All data are expressed as mean ± standard error of the mean (SEM). All statistical analyses were performed using GraphPad Prism Version 8.0 (GraphPad Software Inc., San Diego, CA, USA). Values of *p* < 0.05 were considered statistically significant. All experiments were repeated at least three times.

## Results

### General condition and organ weights of rats

**Figure 1:**
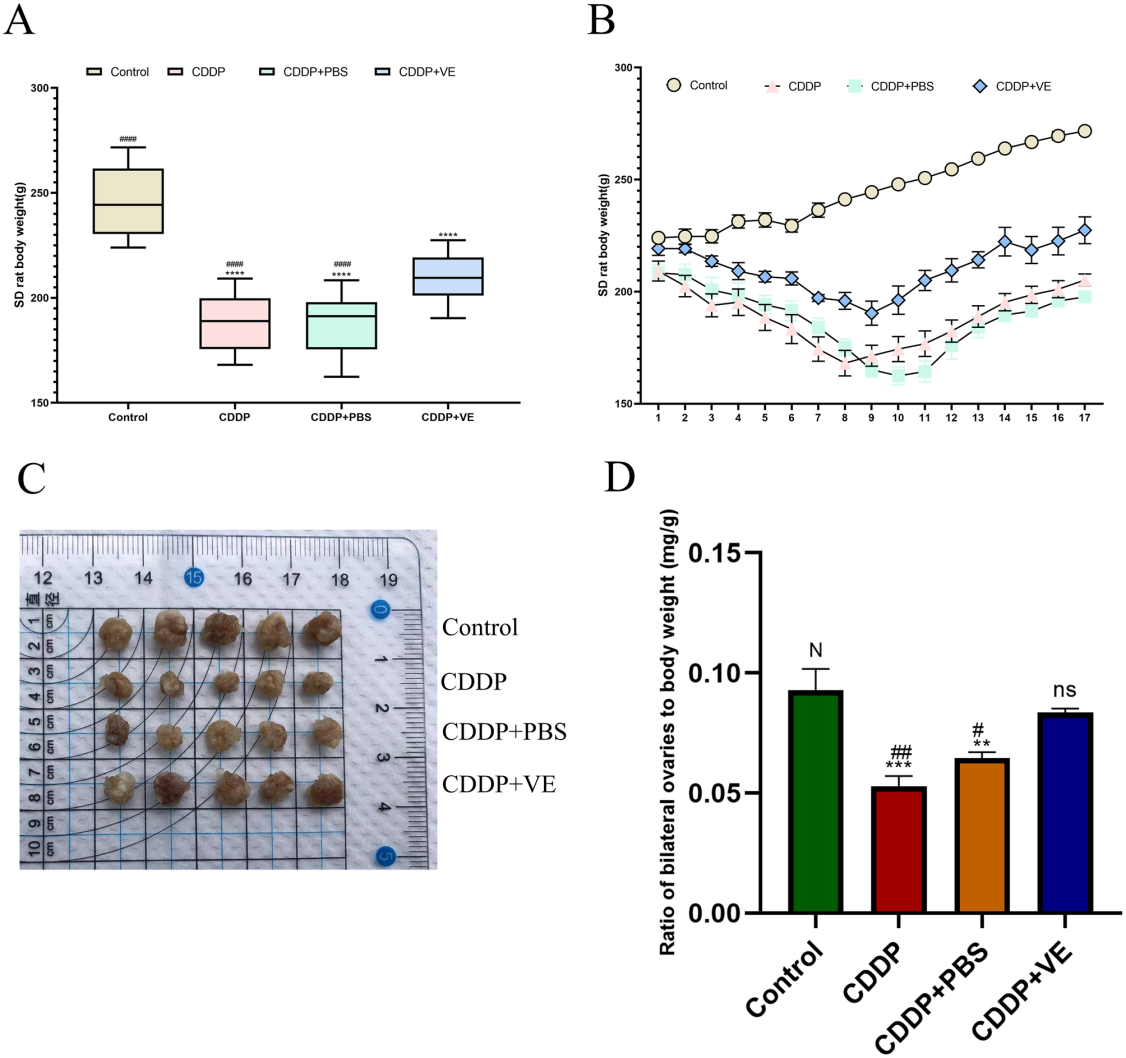
Body and ovary weights of rats. (**A**, **B**) Body weight changes trend of rats in each group. (**C**, **D**) Ovarian size and the ratio of bilateral ovaries to body weight in each group. Data are expressed as the means ± SEM. n = 20, ns, * versus control, *P < 0.05, **P < 0.01, ***P < 0.001 and ****P < 0.0001, ns indicates no statistical significance. N, # versus CDDP + VE, # *P* < 0.05, ## *P* < 0.01, ###*P* < 0.001 and #### *P* < 0.0001, N indicates no statistical significance.

During CDDP administration, rats in all groups except the control exhibited decreased activity, anorexia, rough yellow fur, and dry stools, and some of the rats developed hematuria. Body weight changes after CDDP administration are shown in Fig. 1A,B. The control group steadily gained weight during the experimental period, while the CDDP, CDDP + PBS, and CDDP + VE groups lost weight during the administration period and gradually gained weight after discontinuation of the drug, with the CDDP + VE group losing less weight than the CDDP, and CDDP + PBS group. The body weights of the other three groups at the end of the experiment were statistically significantly different when compared to the control group, as were the CDDP, and CDDP + PBS versus CDDP + VE treatment groups.

The changes in ovary size and weight index in each group after harvesting at the end of the experiment are shown in Fig. 1C,D. Ovarian size was significantly reduced in the CDDP and CDDP + PBS groups compared to the control group, but increased in the CDDP + VE group compared to these two groups (Fig. 1C). The dual ovary weight index results showed significant differences among the control, CDDP, CDDP + PBS, and CDDP + VE groups. Compared with the control group, the ratio of ovary to body weight in the CDDP, and CDDP + PBS group was significantly lower, whereas the ratio in the CDDP + VE group decreased slightly. However, there was also a significant difference in the ratio between the CDDP, and CDDP + PBS groups compared with the CDDP + VE treated group (Fig. 1D).

### Effects of CDDP and ferroptosis inhibitor vitamin E on ovarian function and fertility in rats

#### Effects of CDDP and VE on ovarian function in rats

**Figure 2:**
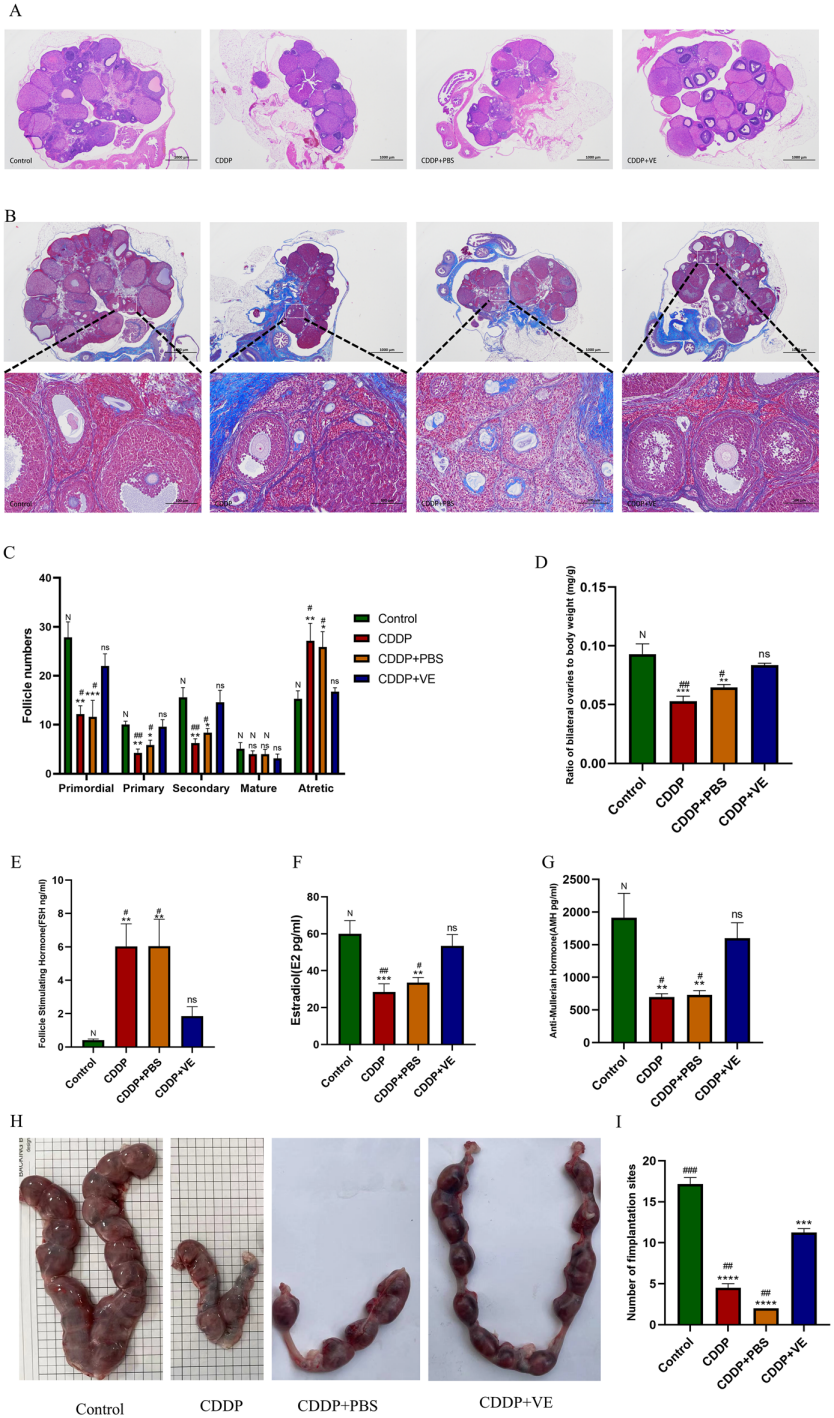
Effects of CDDP and ferroptosis inhibitor Vitamin E on ovarian function and fertility in rats. (**A**, **C**) HE staining of the ovarian tissue structure in each group and the statistical results of the number of follicles in different developmental stages. (**B**, **D**) Masson trichrome staining of the ovarian tissue in each group observed under a microscope. (**E**, **F**, **G**) Serum FSH, E2 and AMH levels in each group. (**H**) Gross observation of uterine litter size of rats in each group. (I) Summary of embryo numbers at implantation sites of each group. The score of stained Masson trichrome Staining was quantitated using ImageJ software. Data are expressed as the means ± SEM. ns, * versus control, *P < 0.05, **P < 0.01, ***P < 0.001 and ****P < 0.0001, ns indicates no statistical significance. N, # versus CDDP + VE, # *P* < 0.05, ## *P* < 0.01, ###*P* < 0.001 and #### *P* < 0.0001, N indicates no statistical significance.

We explored whether the establishment of a rat model of POI was successful in four aspects, including estrous cycle monitoring, follicle counts, ovarian tissue fibrosis and serum sex hormone levels, as well as changes in ovarian function in POI rats after treatment with the ferroptosis inhibitor VE. The results showed that the estrous cycles of rats in the control group were regular, those in the CDDP and CDDP + PBS groups were disordered and irregular during the experimental period, whereas those in the VE treated group were relatively regular, although prolonged (Supplemental Figure. 1). The results of serum hormones showed that compared with the control group, the levels of E2 and AMH in the rats in the CDDP and CDDP + PBS groups were significantly lower, while the levels of FSH were significantly higher. The differences were statistically significant, indicating successful establishment of the POI animal model. However, rats treated with VE had significantly higher serum E2 and AMH levels and decreased FSH secretion compared to the CDDP, and CDDP + PBS group (Fig. 2E,F,G).

HE and Masson trichrome staining of ovarian tissue showed that the control group had a large number of healthy follicles at all stages, including primordial, primary, secondary and mature follicles, as well as a small number of atretic follicles and a small area of blue collagen fibers. In contrast, the ovaries in the CDDP and CDDP + PBS groups were atrophic, with a significant increase in the number of atretic follicles and blue fibrotic area, but a decrease in the number of follicles at other different stages of development. After treatment with VE, the number of primordial, primary, and secondary follicles in the CDDP + VE group was significantly higher than that in the CDDP and CDDP + PBS groups, while the number of atretic follicles and collagen fibrosis area were significantly decreased, but there was no significant difference in the number of mature follicles among the groups (Fig. 2A,B,C,D). These results indicate that CDDP causes impaired follicle development and ovarian tissue fibrosis in rats, while VE treatment helps to restore ovarian damage due to CDDP.

#### Effects of CDDP and VE on fertility in POI rats

The embryo implantation rate and pregnancy rate of rats in each group were determined. As shown in Fig. 2H,I, the implantation rate of embryos in the CDDP and PBS treatment groups was significantly lower than that in the control group, whereas the implantation rate of embryos in POI rats significantly increased in the VE treatment group. In addition, the results in Table 1 show that pregnancy rates were improved in POI rats after VE treatment. Taken together, the ferroptosis inhibitor VE can help restore fertility in POI rats.

**Table 1:** Effects of CDDP and VE on pregnancy rates in rats.

Group	Pregnancy	Not pregnancy	Pregnancy, rate %(n/n)	*P* value
Control	6	0	100% (6/6)	0.0606^a^
CDDP	2	4	33.33% (2/6)	> 0.9999^b^
CDDP + PBS	1	5	16.67% (1/6)	
CDDP + VE	4	2	66.67% (4/6)	

Pregnancy rate = total number of pregnancy/total number of matings.

^a^Control group versus CDDP group.

^b^CDDP + VE group versus CDDP group.

*P* values were statistically calculated using Fisher’s exact test.

### Effects of CDDP and VE on ovarian granulosa cell (GC) viability in vitro

#### Identification of GCs

**Figure 3:**
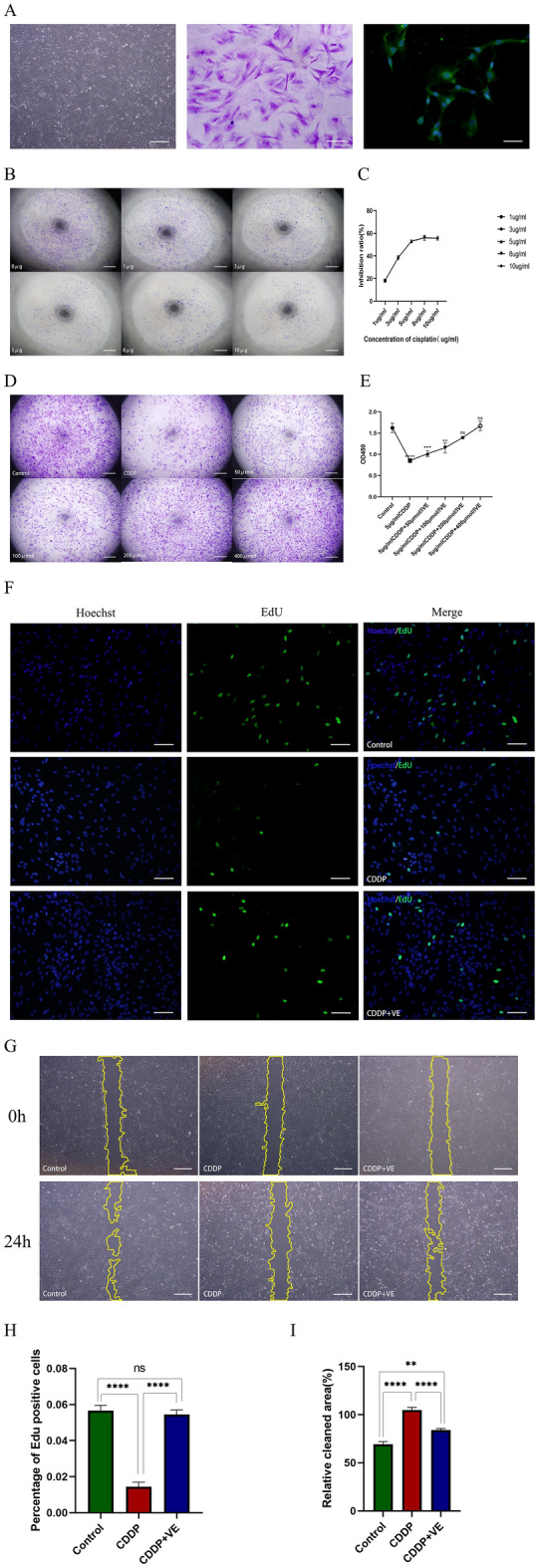
Effects of CDDP and VE on ovarian granulosa cell viability. (**A**) Morphology under light microscopy and crystal violet staining of ovarian granulosa cells (GCs), and identification of FSHR immunofluorescence specifically expressed by GCs. Respectively scale bars are: 500 μm, 100 μm, 50 μm. (**B**, **C**) The inhibition rate of CDDP at different concentrations on the proliferation of GCs was determined by CCK8. Scale bar 500 μm. (**D**, **E**) CCK8 determined the viability of granulosa cells after CDDP injury with different concentrations of VE. Scale bar 500 μm. (F, G, H, I) EdU and wound healing assays were performed to determine the proliferation, migration motility, and repair capacity of the injured GCS after the addition of 200 μmol/l VE to the culture medium. Data are expressed as the means ± SEM. EdU scale bar 100 μm, wound healing scale bar 500 μm. *P < 0.05, **P < 0.01, ***P < 0.001 and ****P < 0.0001, ns indicates no statistical significance.

Primary GCs were extracted from ovarian tissue and cultured for 48 h. Under light microscopy, the cells were spindle-shaped, the nuclei were large and round, and the cells grew in aggregates (Fig. 3A). FSHR immunocytochemical staining is specific for GCs. FSHR positive staining was localized in the cell membrane, with green staining and dark blue staining in the nucleus. Microscopically, the FSHR positive rate was > 95% (Fig. 3A), thereby indicating that the purity of the isolated and cultured GCs was more than 95%.

#### Effects of CDDP and VE on GCs viability

To identify a suitable concentration of CDDP to inhibit GCs proliferation and VE treatment of injured GCs, the viability of GCs at different concentrations was measured using the CCK-8 kit according to previous reports. When the concentration of CDDP was 5 μg/mL, the cell inhibition rate was approximately 53.05%, and the observation of cell Crystal Violet staining in 96-well plates was consistent with that of the CCK-8 assay (Fig. 3B, .C). When the VE concentration was 200 μmol/L, cell viability was 86.25% and at 400 μmol/L, the cell viability was 106.90% and there was no significant difference between the two concentrations. The observation of cell crystal violet staining in 96-well plates was consistent with the CCK-8 assay (Fig. 3D,E). Therefore, 5 μg/mL CDDP and 200 μmol/L VE were used to perform the EdU and wound healing assay. The proliferation of GCs was obviously decreased after CDDP injury. With longer reaction times, cell morphology changed, cell number was significantly reduced, and migration at the scratch was not obvious, whereas VE reversed the damaging effect of CDDP on GCs (Fig. 3F-I). These results showed that CDDP causes GCs damage, which was reversed by the ferroptosis inhibitor VE.

### Mechanism of action of ferroptosis in mediating POI induced by CDDP

#### Ferroptosis is present in POI caused by CDDP

**Figure 4:**
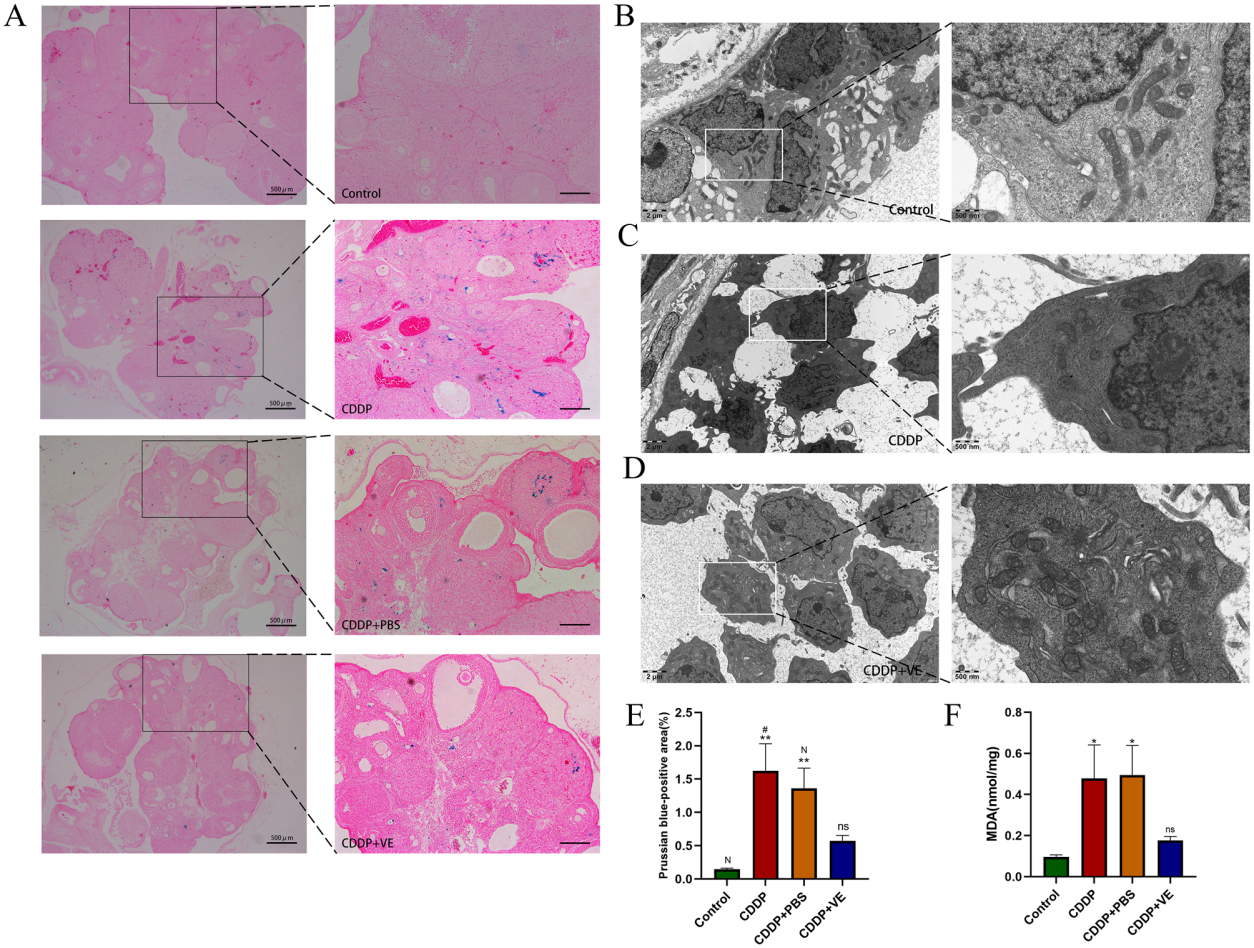
Effects of CDDP and VE on ferroptosis in ovarian tissue. (**A**, **E**) Perls staining of ovarian tissues in each group, and Fe^3+^ blue positive areas were calculated using image J computing software. Scale bar 200 μm. (**B**, **C**, **D**) Transmission electron microscopy was used to observe the mitochondrial ultrastructure in GCs in the ovary tissues of each group. (**F**) MDA expression levels in ovarian tissues of each group. Data are expressed as the means ± SEM. n = 20, ns, * versus control, *P < 0.05, **P < 0.01, ***P < 0.001 and ****P < 0.0001, ns indicates no statistical significance. N, # versus CDDP + VE, # *P* < 0.05, ## *P* < 0.01, ###*P* < 0.001 and #### *P* < 0.0001, N indicates no statistical significance.

As shown in Fig. 4B, the number of GCs in the control group was greater and the cells were closely arranged. The mitochondria were numerous and well distributed in the cells, and the mitochondrial bilateral membranes and mitochondrial cristae were clear. The number of GCs decreased and the intercellular spaces were larger in the CDDP group. Mitochondria in this group were reduced in number and size, with flattened mitochondrial cristae, increased membrane density, and some outer mitochondrial membranes were ruptured (Fig. 4C). Whereas in the VE group, the number of cells and mitochondria increased significantly, mitochondrial volume increased, and mitochondrial cristae were clear, but the density of the mitochondrial membrane did not change significantly (Fig. 4D). The results of MDA expression level assays suggested that lipid peroxide levels were significantly increased in the CDDP and CDDP + PBS groups and were reduced by VE treatment (Fig. 4F). Perls staining results showed that the Fe^3+^ blue positive area in the ovaries of the CDDP and CDDP + PBS groups was significantly increased compared with that in the control group, and the area of iron deposition was significantly reduced after VE treatment (Fig. 4A, .E). The above results suggested that ferroptosis occurred in POI and that VE inhibited ferroptosis in POI.

#### Bioinformatics data analysis suggested an association between ferroptosis and POI.

Our search of the GO database with the term “primary ovarian insufficiency” identified genes differentially expressed in POI, with a subset of genes strongly associated with ferroptosis (GSE178302). Further KEGG and GO enrichment analysis of these differential genes revealed that the differential genes in POI and as a key gene in the ferroptosis pathway, acetyl CoA synthetase long chain family member 4 (ACSL4) may play a key role in CDDP-induced POI (Supplemental Figure 2). 15-lipoxygenase-1 (ALOX15) is a downstream gene in the pathway involved in membrane lipid peroxidation by ACSl4, and the ferroptosis inhibitor VE repressed ALOX15 expression in this pathway and reversed the occurrence of ferroptosis, whereas SLC7A11 and GPX4 were key genes in the suppression of ferroptosis (Supplemental Figure 2).

#### Protein and RNA expression level changes of ferroptosis-related molecules in rat ovarian tissues and GCs.

**Figure 5:**
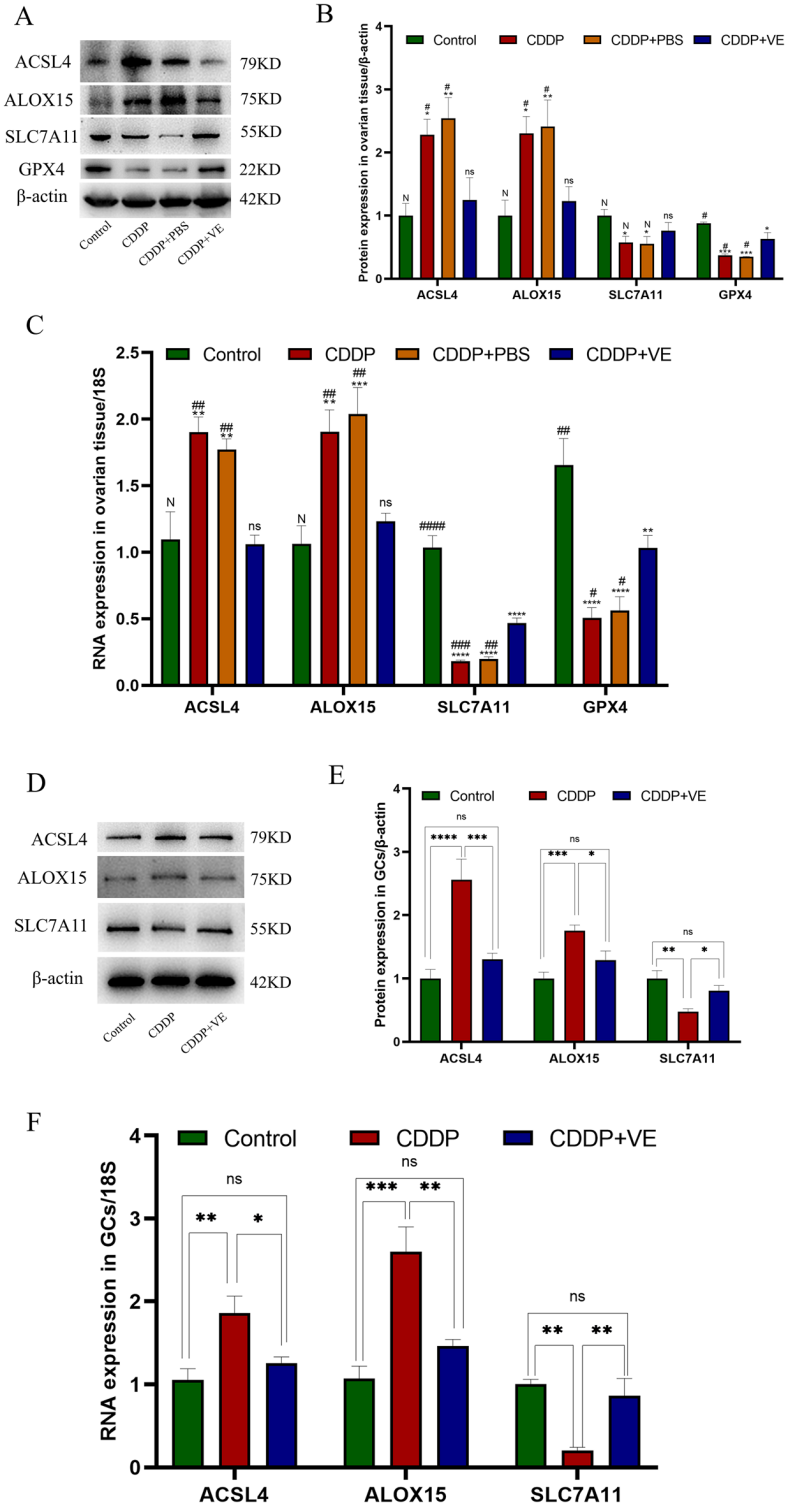
Protein and RNA expression level changes of ferroptosis related molecules in ovarian tissues and GCs of each group. (**A**, **D**) Western blot bands of ferroptosis related molecules in ovarian tissue and GCs. One representative blot from three independent experiments is shown, β-actin was used as the loading control. (**B**) Quantitation on protein expression of ACSL4, ALOX15, SLC7A11, and GPX4 in ovarian tissues. (**C**) The RNA expression of ACSL4, ALOX15, SLC7A11 , and GPX4 by qRT-PCR analysis in ovarian tissues. (**E**) Quantitation on protein expression of ACSL4, ALOX15, SLC7A11 in GCs. (**F**) The RNA expression of ACSL4, ALOX15, SLC7A11 by qRT-PCR analysis in GCs. 18S was used as an internal control. Data are expressed as the means ± SEM. ns, * versus control, *P < 0.05, **P < 0.01, ***P < 0.001 and ****P < 0.0001, ns indicates no statistical significance. N, # versus CDDP + VE, # *P* < 0.05, ## *P* < 0.01, ###*P* < 0.001 and #### *P* < 0.0001, N indicates no statistical significance.

**Figure 6:**
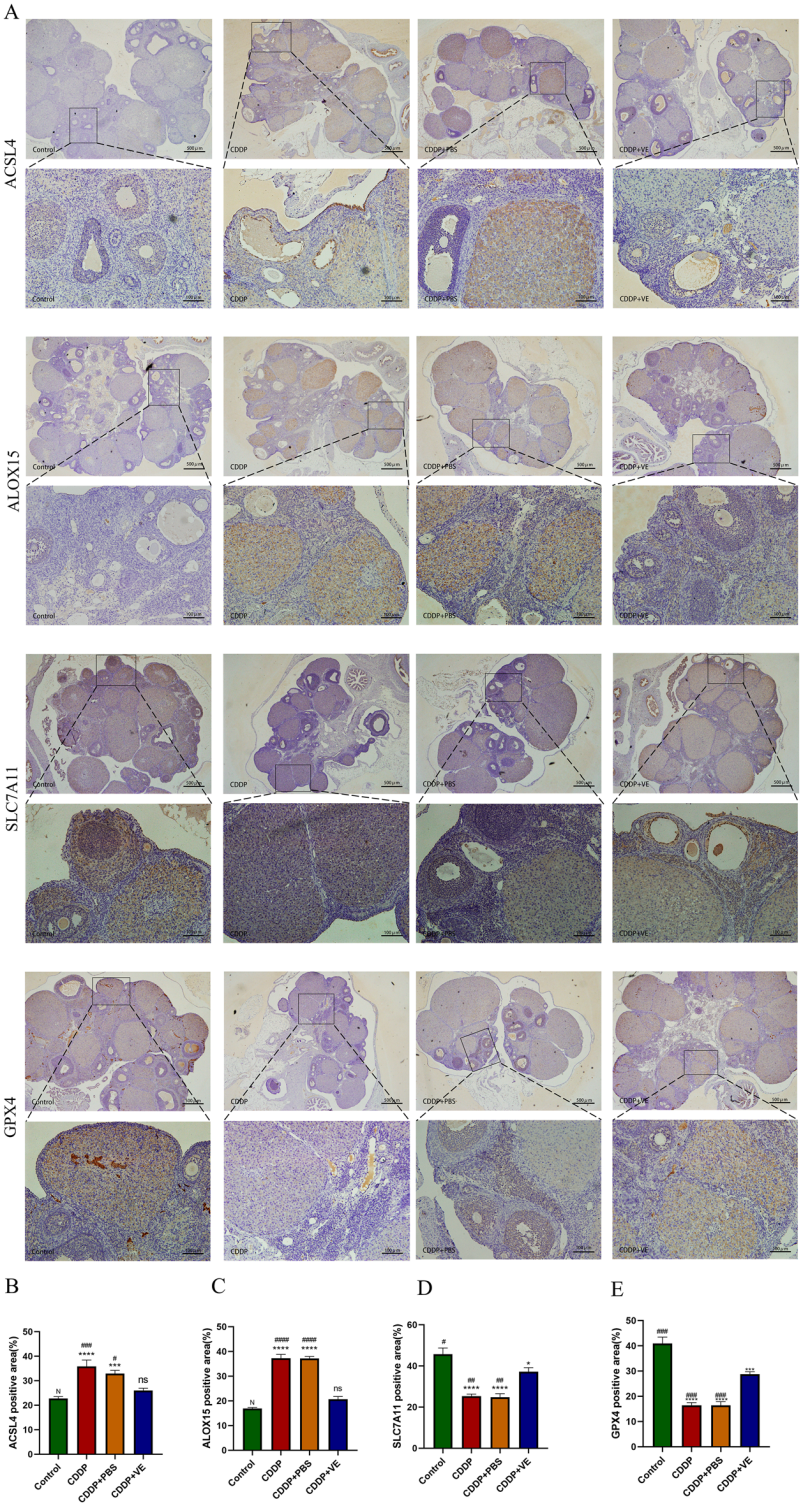
Immunohistochemical staining of ferroptosis related molecules in ovarian tissues. (**A**) ACSL4, ALOX15, SLC7A11 and GPX4 immunohistochemical micrographs of ovarian tissue from each group. (**B**, **C**, **D**, **E**) The protein expressions (area of brown yellow positive area) of ACSL4, ALOX15, SLC7A11 and GPX4 in the immunohistochemical photographs were quantified using Image J software. Data are expressed as the means ± SEM. n = 20, ns, * versus control, *P < 0.05, **P < 0.01, ***P < 0.001 and ****P < 0.0001, ns indicates no statistical significance. N, # versus CDDP + VE, # *P* < 0.05, ## *P* < 0.01, ###*P* < 0.001 and #### *P* < 0.0001, N indicates no statistical significance.

Western blot analysis of protein extracted from ovarian tissue in each group showed higher expression of ACSl4 and ALOX15 and lower expression of SLC7A11 and GPX4 in the POI and PBS treated rats compared with control rats, whereas treatment with the ferroptosis inhibitor VE resulted in lower expression of ACSL4 and ALOX15 and higher expression of SLC7A11 and GPX4 with statistically significant differences (Fig. 5A,B). To further verify the protein expression differences in ferroptosis-related molecules in each group, immunohistochemical staining was performed on ovarian tissue sections from each group. The staining results were quantitatively analyzed using Image J software, and the statistical results were generally consistent with those of Western blot analysis (Fig. 6A,B,C,D,E). The RNA expression of ferroptosis-related molecules in each group was determined by qRT-PCR, and the results were consistent with the protein expression trend (Fig. 5C). These experimental findings suggested that ferroptosis was increased in the ovarian tissue of POI rats compared with controls and was inhibited by VE.

The expression of ferroptosis-related molecules in GCs showed that compared with the control group, the protein and RNA expression of ACSL4 and ALOX15 was significantly increased and the expression of SLC7A11 was significantly decreased following GCs injury by 5 μg/mL CDDP. However, after adding 200 μmol/L VE to the medium, the protein and RNA levels of ACSL4, ALOX15 and SLC7A11 were reversed (Fig. 5D,E,F). These results were consistent with the expression of related molecules in ovarian tissue.

## Discussion

Our experimental results confirmed that ferroptosis mediated CDDP-induced ovarian tissue fibrosis in rats, resulting in POI. The reproductive toxic effects of CDDP on the ovary could be reversed using the ferroptosis inhibitor VE. The global incidence of POI is 1% before the age of 40 years and 0.1% before the age of 30 years, whereas in women aged < 20 years the incidence is 0.01%^23^. The treatment of POI and the restoration of fertility, are intractable problems long faced by obstetricians and gynecologists worldwide. The conventional treatment modalities are: hormone supplement therapy, gene therapy, stem cell therapy, intra-ovarian injection of autologous platelet rich plasma therapy, oocyte donation, etc^24–27^. Previous results from these studies suggest that some of these treatment modalities have suboptimal outcomes, and some, due to ethical, expense, and other issues, ultimately fail to achieve the desired outcomes for clinicians and patients. Clinicians’ research on the pathogenic mechanisms and treatment modalities of POI have continued, and with the proposal of the concept of ferroptosis as a novel programmed cell death modality, the significant research findings in fibrosis-related diseases have attracted the attention of scientific researchers, and the basic pathological changes in POI involve ovarian tissue fibrosis, which provided a theoretical basis for our study.

Chemotherapy is an important factor causing ovarian hypofunction in young women, and CDDP is the first-line chemotherapeutic drug for many tumors. Studies have shown that CDDP can cause significant lipid peroxidation and promote cancer cell ferroptosis^28,29^, and numerous studies have previously reported the use of CDDP to establish animal models of POI^30,31^. Therefore, our study first confirmed the successful establishment of a rat model of POI using CDDP, and impaired ovarian function was significantly improved in rats treated with VE, with statistically significant changes in the number of follicles, the degree of ovarian tissue fibrosis, as well as serum sex hormone levels. The results of the fertility monitoring study showed that the number of pregnancies and litters were significantly higher in VE treated rats compared to the CDDP group, but there was no statistically significant difference, possibly due to the smaller sample size in the fertility assay and the shorter observation time. In addition, during the observation period, we found that control rats became pregnant soon after cage closure, whereas the CDDP and CDDP + PBS groups exhibited their first pregnancies between 3 and 6 months after withdrawal, and the VE group exhibited their first pregnancies at 2–4 months. Masson staining of ovarian tissue sections demonstrated a significant improvement in fibrosis in the VE group. These results tentatively indicated that CDDP caused POI with a long duration of toxic effects on ovarian function and that inhibitors of ferroptosis mitigated the reproductive toxic effects of CDDP.

We next investigated whether ferroptosis was present in POI caused by CDDP. Using TEM to investigate the ultrastructure of GCs in ovarian tissue, we observed characteristic mitochondrial changes indicative of ferroptosis in the CDDP group, which improved in the VE treated group. MDA is one of the most important products of membrane lipid peroxidation, and its production can aggravate cell membrane damage. Therefore, MDA is a commonly used indicator of lipid peroxidation, which indirectly determines the degree to which ferroptosis occurs, and detection of the expression level of MDA reflected the degree of membrane lipid peroxidation in many studies^32,33^. Our results also suggested that MDA expression levels were significantly increased in the CDDP and CDDP + PBS groups and that this level decreased after VE treatment. Ferroptosis is mainly programmed cell death induced by iron overload; therefore, we stained ovarian tissue to assess Perls ferric iron deposits and showed that the area of blue iron deposits was significantly increased in the CDDP and CDDP + PBS groups, but decreased in the VE group. The above experimental results may provide preliminary evidence that ferroptosis is present in POI induced by CDDP, and the ferroptosis inhibitor VE can either inhibit the progression of POI or improve impaired ovarian function in POI.

To further understand the mechanism by which ferroptosis mediates POI caused by CDDP, we performed a bioinformatics database search for POI and a subset of the differential genes screened was strongly associated with ferroptosis. ACSl4 is a key gene that induces lipid peroxidation, causing ferroptosis. Together with LPCAT3, it catalyzes arachidonic acid and adrenic acid belonging to the family of polyunsaturated fatty acids (PUFAs) to esterify them into phosphatidylethanolamine, which then acts with different lipoxygenases (such as ALOX15) to produce lipid peroxides, causing changes in the fluidity and permeability of the cell membrane, affecting membrane function and ultimately causing the cell to undergo ferroptosis^34–36^. Some studies have shown that CDDP can significantly increase the expression level of ACSl4, cause lipid peroxidation, and promote cell ferroptosis^28,29,37^. ALOX15 is a non heme, iron containing fatty acid dioxygenase that metabolizes several PUFAs to form biologically active lipid mediators^38^. Several studies have recently shown that ALOX15 can directly oxidize arachidonic acid and adrenic acid in the absence of glutathione peroxidase 4 (GPX4) to generate a ferroptosis signal^39^. ALOX15 mediated cell ferroptosis is observed in many central nervous system (CNS) disorders^40^.

GPX4 is a central factor that inhibits cells from undergoing ferroptosis^41^, and its expression and activity are dependent on cysteine responsible for the synthesis of glutathione (GSH), and cysteine is transported into the cell by the cystine/glutamate antiporter (System Xc^−^). When SLC7A11, the major functional subunit of system Xc^−^, is deficient or has reduced activity, GSH synthesis is reduced, causing reduced expression and activity of GPX4, resulting in the inability of cells to effectively scavenge lipid peroxides, and finally oxidative damage to the cell membrane, leading to cell ferroptosis^42^. Previous studies have reported a correlation between GSH, GPX4 and ovarian dysfunction; however, few studies have focused on the correlation between SLC7A11 and POI^43–46^. Therefore, we mainly selected SLC7A11 to further validate the association of POI and ferroptosis, but we still validated the expression of GPX4 in animal experiments.

Vitamin E, as a fat soluble vitamin, is one of the most important antioxidants in the body and one of the major inhibitors of ferroptosis^47^. During the onset of ferroptosis, reduced Fe^3+^ on ALOX15 inactivated Fe^2+^ and inhibited ferroptosis by competing with ALOX15 for the PUFA substrate binding site^39^. In several studies, it was shown that a lack of VE can cause severe lipid peroxidation^48^. Dietary VE supplementation during pregnancy prevented the destruction of mouse embryonic fibroblasts and cortical neurons caused by GPX4 knockout and increased the survival of fetal mice^49^. In previous studies of ovarian function, VE due to its antioxidant activity was able to improve Diazinon-induced ovarian toxicity^50^; and improved in vitro maturation rates and blastocyst rates of oocytes isolated from vitrified ovarian tissue^51^. VE levels in women with POI were significantly lower than those in women with normal menstrual cycles^52^. Supplementation with VE can increase the AMH index, antral follicle count (AFC) and mean ovarian volume (MOV) in women with POI^53^. Therefore, we selected VE as a ferroptosis inhibitor to investigate the mechanism of action by which ferroptosis mediates POI caused by CDDP.

We measured the protein and gene expression levels of the ferroptosis-related molecules ACSl4, ALOX15, SLC7A11, and GPX4 in each group. The results were consistent with our theory, where ACSl4 and ALOX15, as genes promoting ferroptosis, were significantly higher in the CDDP group and decreased after treatment with the ferroptosis inhibitor VE, whereas both genes exhibited higher levels in the PBS placebo treated group. In contrast, SLC7A11 and GPX4, as suppressor genes, decreased significantly in the CDDP and CDDP + PBS groups and increased in the VE group. We also performed immunohistochemistry on ovarian tissue sections and the results were consistent with the Western blot and qPCR results. These results proved that ferroptosis may mediate the occurrence of POI by CDDP through ACSl4, ALOX15, SLC7A11, and GPX4.

Previous studies have shown that GCs were the largest cell population in ovarian tissue and that proliferation and differentiation of GCs are important for oocyte developmental maturation during follicular maturation^54^. A large number of gap junctions exist between GCs and oocytes through which nutrients and signaling molecules are transported into the oocyte to promote oocyte nuclear versus cytoplasmic maturation^55^. Death of GCs can inhibit oocyte and follicle development, and promote POI triggered by follicular atresia^56^. Therefore, to further validate our experiments, we cultured GCs in vitro. Using different concentrations of CDDP to stimulate GCs, the optimal lesion concentration was experimentally selected using the CCK-8 assay, and according to the same method, the optimal concentration of VE treatment was determined. The optimal therapeutic concentration of VE was further verified by the EdU and wound healing experiments. Determination of ferroptosis-associated molecules was performed using western blotting and qPCR, and the findings were consistent with the in vivo experiments.

The present study had the following limitations: 1. In principle, it is more convincing to use ovarian tissue from POI patients and clinical drug trials for research validation. However, due to ethical issues, collection of human ovarian tissue is difficult, and the many pathogenic factors and individual differences that cause POI can easily lead to inaccurate results. Therefore, the chemotherapeutic agent was chosen as a single stimulus in this study to perform dual validation in both in vitro and in vivo tests to guarantee the accuracy of experimental results as much as possible. In the future, we will select more animal models of POI with other pathogenic factors such as immunization, radiotherapy, and genetics for the investigation of ferroptosis-related mechanisms. Attempts can also be made to collect human ovarian tissue or follicular fluid for research, and to initiate clinical drug trials. 2. As ferroptosis has been poorly studied in POI, this study was only at the exploratory stage, no complete and coherent study of signaling pathways has been performed in relevant experiments, and ferroptosis has only been verified in animal and cellular models of chemotherapy-induced POI, and the likely mechanism of action is regulation of the expression of ACSl4, ALOX15, SLC7A11, and GPX4. In the next step, we will conduct a series of trials to perform further investigations, including studies on the molecules upstream and downstream of coherent signaling pathways, using genetically modified animals and viruses to transfect cells, more cell lines or ferroptosis inhibitors, or ferroptosis inducers to establish POI models. 3. Although our experiments confirmed that GCs ferroptosis was increased in POI, whether ferroptosis predominated, and the association with other cell death modalities that we did not validate, will be investigated in the future. 4. Our experiments used only Masson trichrome staining to analyze blue collagen fiber occupancy to demonstrate that ferroptosis mediates CDDP to cause ovarian tissue fibrosis, triggering POI. Therefore, the findings were weak, and in follow-up studies we will measure the expression levels of fibrosis related signaling pathway molecules following the administration of ferroptosis inhibitors, and monitor ovarian function and related molecular changes after the use of fibrosis related molecular inhibitors, ferroptosis inhibitors, and the combination of the two inhibitors, to further confirm the results of our mechanism study. 5. The small sample size and short observation time in the fertility determination experiment in this study resulted in non-statistically significant results. In subsequent related studies, we intend to expand the sample size and prolong the caging time, observe the number of pregnancies in each rat, the number of litters per time, and observe the fertility of offspring pups to monitor the long-term therapeutic effects.

## Conclusion

In summary, our findings suggested that ferroptosis mediated POI caused by CDDP. The main mechanism may involve regulation of ACSl4, ALOX15, SLC7A11, and GPX4 expression by CDDP to promote ferroptosis in ovarian GCs and cause ovarian dysgenesis and ovarian tissue fibrosis in rats. The ferroptosis inhibitor VE inhibited GCs ferroptosis, repair ovarian function, and improved fertility.

## Supplementary Information

Supplementary Figure 1.

Supplementary Figure 2.

Supplementary Information 3.

## Data Availability

All data presented in the present study are available from the corresponding author on reasonable request.

## References

[1] European society for human R, Embryology guideline group on P O I, Webber, L. et al. ESHRE Guideline: Management of women with premature ovarian insufficiency. *Hum. Reprod.* **31**(5), 926–37 (2016). 10.1093/humrep/dew027 27008889

[2] Cattoni A, Parissone F, Porcari I (2021). Hormonal replacement therapy in adolescents and young women with chemo- or radio-induced premature ovarian insufficiency: Practical recommendations. Blood Rev..

[3] Spears N, Lopes F, Stefansdottir A (2019). Ovarian damage from chemotherapy and current approaches to its protection. Hum. Reprod. Updat..

[4] Bedoschi G, Navarro PA, Oktay K (2016). Chemotherapy-induced damage to ovary: Mechanisms and clinical impact. Futur. Oncol. Lond. Eng..

[5] Fraison E, Crawford G, Casper G (2019). Pregnancy following diagnosis of premature ovarian insufficiency: A systematic review. Reprod. Biomed. Online.

[6] Sarrel PM, Sullivan SD, Nelson LM (2016). Hormone replacement therapy in young women with surgical primary ovarian insufficiency. Fertil. Steril..

[7] Dixon SJ, Lemberg KM, Lamprecht MR (2012). Ferroptosis: An iron-dependent form of nonapoptotic cell death. Cell.

[8] Bertrand RL (2017). Iron accumulation, glutathione depletion, and lipid peroxidation must occur simultaneously during ferroptosis and are mutually amplifying events. Med. Hypotheses.

[9] Galluzzi L, Vitale I, Aaronson SA (2018). Molecular mechanisms of cell death: recommendations of the Nomenclature Committee on Cell Death 2018. Cell Death Differ..

[10] Stockwell BR, Friedmann Angeli JP, Bayir H (2017). Ferroptosis: A regulated cell death nexus linking metabolism, redox biology, and disease. Cell.

[11] Yu Y, Jiang L, Wang H (2020). Hepatic transferrin plays a role in systemic iron homeostasis and liver ferroptosis. Blood.

[12] Wang L, Zhang Z, Li M (2019). P53-dependent induction of ferroptosis is required for artemether to alleviate carbon tetrachloride-induced liver fibrosis and hepatic stellate cell activation. IUBMB Life.

[13] Kong Z, Liu R, Cheng Y (2019). Artesunate alleviates liver fibrosis by regulating ferroptosis signaling pathway. Biomed. Parmacotherapy.

[14] Cheng H, Feng D, Li X (2021). Iron deposition-induced ferroptosis in alveolar type II cells promotes the development of pulmonary fibrosis. Biochim. Biophys. Acta.

[15] Li X, Zhuang X, Qiao T (2019). Role of ferroptosis in the process of acute radiation-induced lung injury in mice. Biochem. Biophys. Res. Commun..

[16] Liu J, Zhang M, Qin C (2022). Resveratrol attenuate myocardial injury by inhibiting ferroptosis via inducing KAT5/GPX4 in myocardial infarction. Front. Pharmacol..

[17] Zhang Y, Mou Y, Zhang J (2022). Therapeutic implications of ferroptosis in renal fibrosis. Front. Mol. Biosci..

[18] Cui L, Bao H, Liu Z (2020). hUMSCs regulate the differentiation of ovarian stromal cells via TGF-β(1)/Smad3 signaling pathway to inhibit ovarian fibrosis to repair ovarian function in POI rats. Stem Cell Res. Ther..

[19] Guo J, Xu B, Han Q (2018). Ferroptosis: A novel anti-tumor action for cisplatin. Cancer Res. Treat..

[20] Liu Q, Wang K (2019). The induction of ferroptosis by impairing STAT3/Nrf2/GPx4 signaling enhances the sensitivity of osteosarcoma cells to cisplatin. Cell Biol. Int..

[21] Cora MC, Kooistra L, Travlos G (2015). Vaginal cytology of the laboratory rat and mouse: Review and criteria for the staging of the estrous cycle using stained vaginal smears. Toxicol. Pathol..

[22] Ozcelik B, Turkyilmaz C, Ozgun MT (2010). Prevention of paclitaxel and cisplatin induced ovarian damage in rats by a gonadotropin-releasing hormone agonist. Fertil. Steril..

[23] Kokcu A (2010). Premature ovarian failure from current perspective. Gynecol. Endocrinol. Off. J. Int. Soc. Gynecol. Endocrinol..

[24] Ishizuka B (2021). Current understanding of the etiology, symptomatology, and treatment options in premature ovarian insufficiency (POI). Front. Endocrinol..

[25] Cakiroglu Y, Saltik A, Yuceturk A (2020). Effects of intraovarian injection of autologous platelet rich plasma on ovarian reserve and IVF outcome parameters in women with primary ovarian insufficiency. Aging.

[26] Li Z, Zhang M, Tian Y (2021). Mesenchymal stem cells in premature ovarian insufficiency: Mechanisms and prospects. Front. Cell Dev. Biol..

[27] Atabiekov I, Hobeika E, Sheikh U (2018). The role of gene therapy in premature ovarian insufficiency management. Biomedicines.

[28] Orlando UD, Castillo AF, Medrano MAR (2019). Acyl-CoA synthetase-4 is implicated in drug resistance in breast cancer cell lines involving the regulation of energy-dependent transporter expression. Biochem. Pharmacol..

[29] Sha R, Xu Y, Yuan C (2021). Predictive and prognostic impact of ferroptosis-related genes ACSL4 and GPX4 on breast cancer treated with neoadjuvant chemotherapy. EBioMedicine.

[30] Lu X, Bao H, Cui L (2020). hUMSC transplantation restores ovarian function in POI rats by inhibiting autophagy of theca-interstitial cells via the AMPK/mTOR signaling pathway. Stem Cell Res. Ther..

[31] Zhang S, Zhu D, Li Z (2021). A stem cell-derived ovarian regenerative patch restores ovarian function and rescues fertility in rats with primary ovarian insufficiency. Theranostics.

[32] Lai Y, Dong J, Wu Y (2022). Lipid peroxides mediated ferroptosis in electromagnetic pulse-induced hippocampal neuronal damage via inhibition of GSH/GPX4 axis. Int. J. Mol. Sci..

[33] Li P, Lin Q, Sun S (2022). Inhibition of cannabinoid receptor type 1 sensitizes triple-negative breast cancer cells to ferroptosis via regulating fatty acid metabolism. Cell Death Dis..

[34] Zou Y, Henry WS, Ricq EL (2020). Plasticity of ether lipids promotes ferroptosis susceptibility and evasion. Nature.

[35] Chu B, Kon N, Chen D (2019). ALOX12 is required for p53-mediated tumour suppression through a distinct ferroptosis pathway. Nat. Cell Biol..

[36] Doll S, Proneth B, Tyurina YY (2017). ACSL4 dictates ferroptosis sensitivity by shaping cellular lipid composition. Nat. Chem. Biol..

[37] He F, Huang X, Wei G (2022). Regulation of ACSL4-catalyzed lipid peroxidation process resists cisplatin ototoxicity. Oxid. Med. Cell. Longev..

[38] Çolakoğlu M, Tunçer S, Banerjee S (2018). Emerging cellular functions of the lipid metabolizing enzyme 15-Lipoxygenase-1. Cell Prolif..

[39] Kagan VE, Mao G, Qu F (2017). Oxidized arachidonic and adrenic PEs navigate cells to ferroptosis. Nat. Chem. Biol..

[40] Gao S, Zhou L, Lu J (2022). Cepharanthine attenuates early brain injury after subarachnoid hemorrhage in mice via inhibiting 15-lipoxygenase-1-mediated microglia and endothelial cell ferroptosis. Oxid. Med. Cell. Longev..

[41] Yang WS, SriRamaratnam R, Welsch ME (2014). Regulation of ferroptotic cancer cell death by GPX4. Cell.

[42] Chen X, Kang R, Kroemer G (2021). Broadening horizons: The role of ferroptosis in cancer. Nat. Rev. Clin. Oncol..

[43] Chen Y, Fan X, Ma K (2022). Bushen Culuan decoction ameliorates premature ovarian insufficiency by acting on the Nrf2/ARE signaling pathway to alleviate oxidative stress. Front. Pharmacol..

[44] Ma M, Chen XY, Li B (2017). Melatonin protects premature ovarian insufficiency induced by tripterygium glycosides: Role of SIRT1 [J]. Am. J. Transl. Res..

[45] Li F, Wang Y, Xu M (2022). Single-nucleus RNA Sequencing reveals the mechanism of cigarette smoke exposure on diminished ovarian reserve in mice. Ecotoxicol. Environ. Saf..

[46] Ding J, Zhao Q, Zhou Z (2022). Huayu Jiedu Fang protects ovarian function in mouse with endometriosis iron overload by inhibiting ferroptosis. Evid. Complement. Altern. Med. eCAM.

[47] Qi Y, Zhang X, Wu Z (2022). Ferroptosis regulation by nutrient signalling. Nutr. Res. Rev..

[48] Hambright WS, Fonseca RS, Chen L (2017). Ablation of ferroptosis regulator glutathione peroxidase 4 in forebrain neurons promotes cognitive impairment and neurodegeneration. Redox. Biol..

[49] Carlson BA, Tobe R, Yefremova E (2016). Glutathione peroxidase 4 and vitamin E cooperatively prevent hepatocellular degeneration. Redox. Biol..

[50] Sargazi Z, Reza Nikravesh M, Jalali M (2019). The protective effect of vitamin E on rats' ovarian follicles following an administration of diazinon: An experimental study. Int. J. Reprod. Biomed..

[51] Farzollahi M, Tayefi-Nasrabadi H, Mohammadnejad D (2016). Supplementation of culture media with vitamin E improves mouse antral follicle maturation and embryo development from vitrified ovarian tissue. J. Obstet. Gynaecol. Res..

[52] Ma L, Chen G, Xu W (2021). The relationship between vitamin E level and premature ovarian insufficiency. J. Obstet. Gynaecol. Res..

[53] Safiyeh FD, Mojgan M, Parviz S (2021). The effect of selenium and vitamin E supplementation on anti-Mullerian hormone and antral follicle count in infertile women with occult premature ovarian insufficiency: A randomized controlled clinical trial. Complement. Ther. Med..

[54] Tu J, Chen Y, Li Z (2020). Long non-coding RNAs in ovarian granulosa cells. J. Ovarian Res..

[55] Yao J, Huang R, Li M (2021). PTEN expression in human granulosa cells is associated with ovarian responses and clinical outcomes in IVF. Reprod. Sci. Thousand Oaks Calif.

[56] Wang R, Wang W, Wang L (2022). FTO protects human granulosa cells from chemotherapy-induced cytotoxicity. Reprod. Biol. Endocrinol. RB&E.

