# Supplementary material for: Mechanism of ferroptosis in a rat model of premature ovarian insufficiency induced by cisplatin
Source: Sci Rep. 2023 Mar 17;13:4463. doi: 10.1038/s41598-023-31712-7 (PMC10023701; doi:10.1038/s41598-023-31712-7)

**WB image for statistics*: Ovarian tissue***

β -actin ALOX15 Marker/control/CDDP/PBS/VE/ Marker/control/CDDP/PBS/VE/ Marker/control/CDDP/PBS/VE


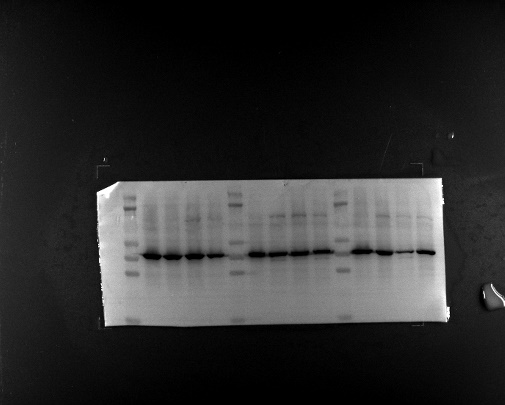

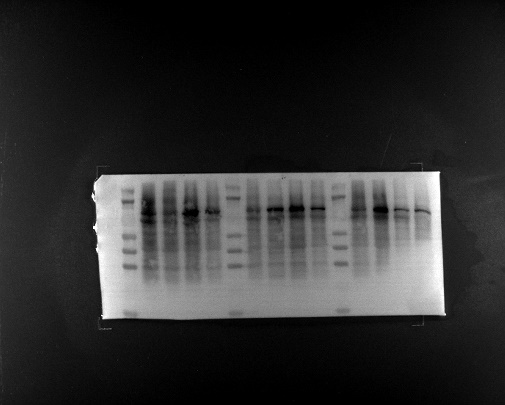


ALOX15+β -actin Marker/control/CDDP/PBS/VE SLC7A11 Marker/control/CDDP/PBS/VE


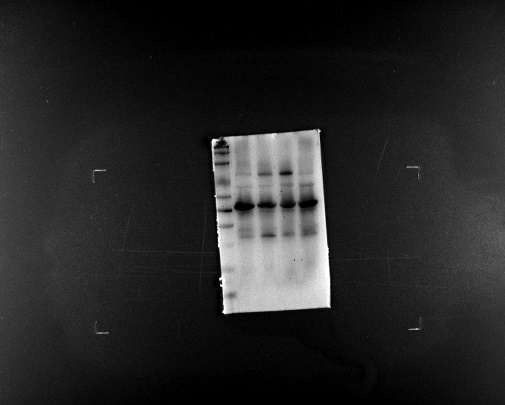

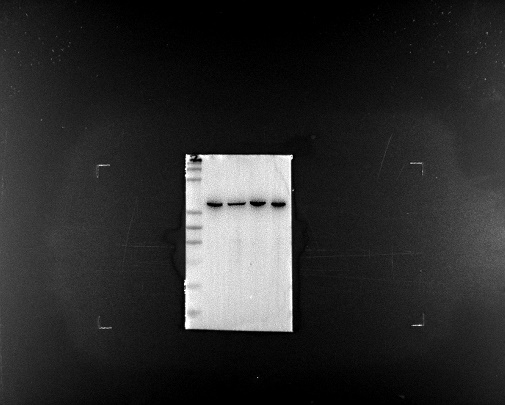


β -actin ACSL4 Marker/control/CDDP/PBS/VE


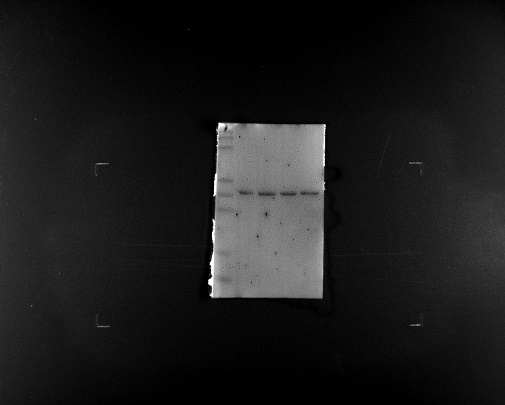

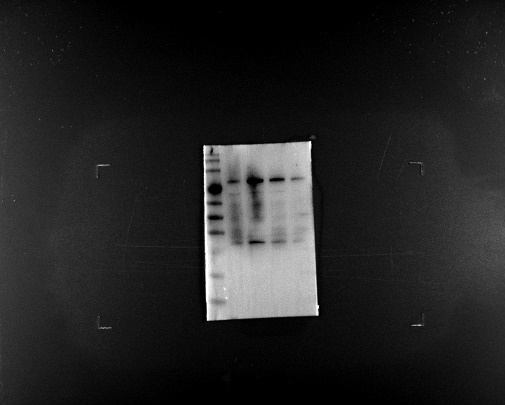


SLC7A11+β -actin Marker/control/CDDP/PBS/VE GPX4 Marker/control/CDDP/PBS/VE


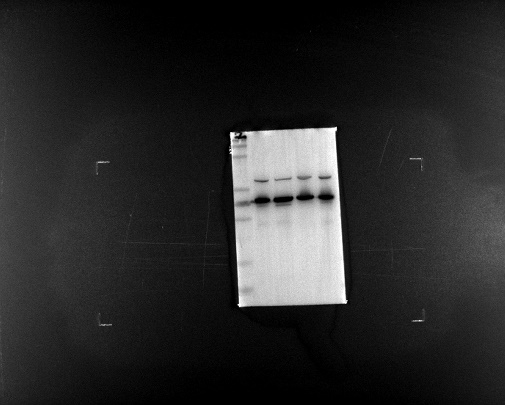

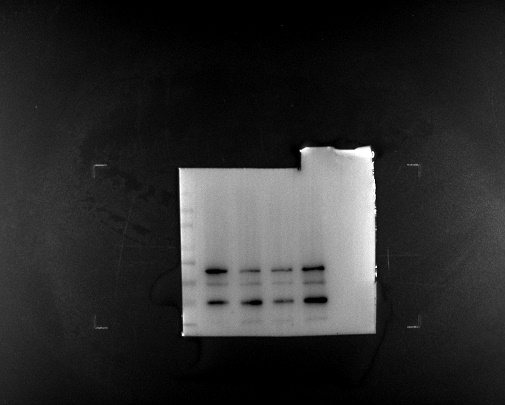


GPX4VE/PBS/CDDP/control/Marker GPX4VE/PBS/CDDP/control/Marker


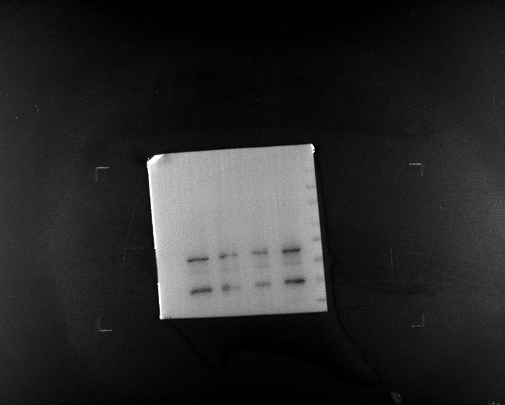

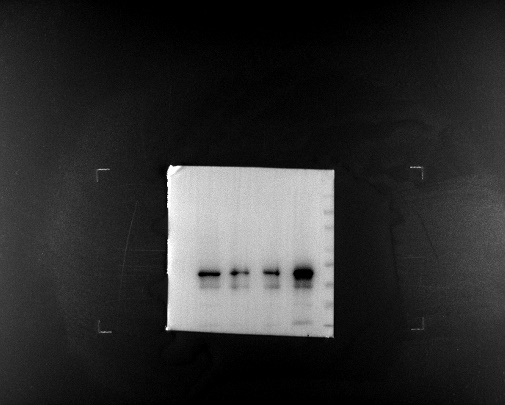


β -actin ACSL4 Marker/control/CDDP/PBS/VE/ Marker/control/CDDP/PBS/VE/ Marker/control/CDDP/PBS/VE


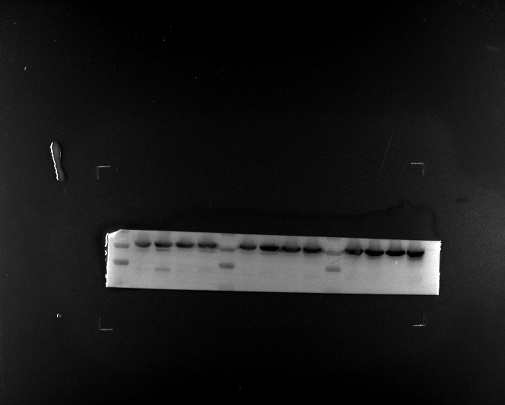

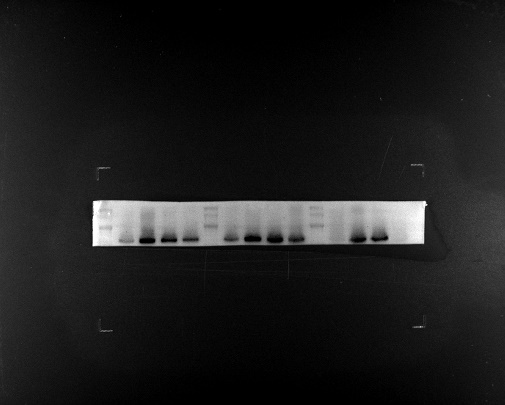


β -actin SLC7A11 Marker/control/CDDP/PBS/VE/ Marker/control/CDDP/PBS/VE/ Marker/control/CDDP/PBS/VE


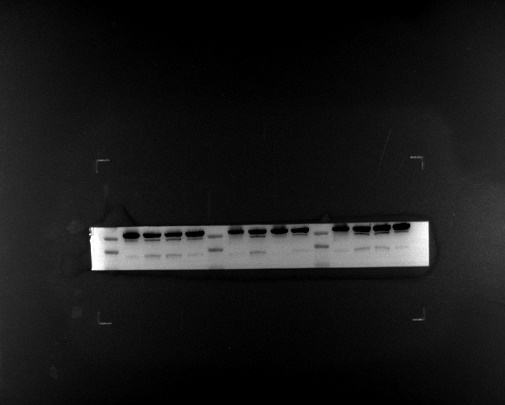

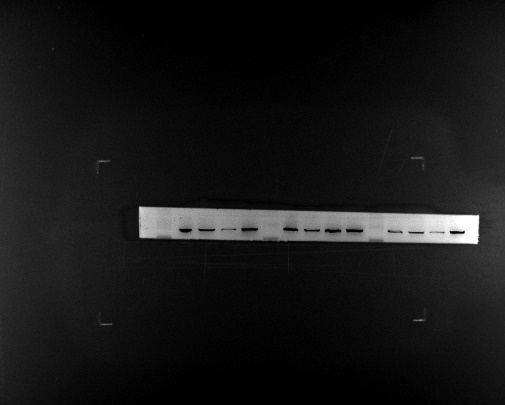


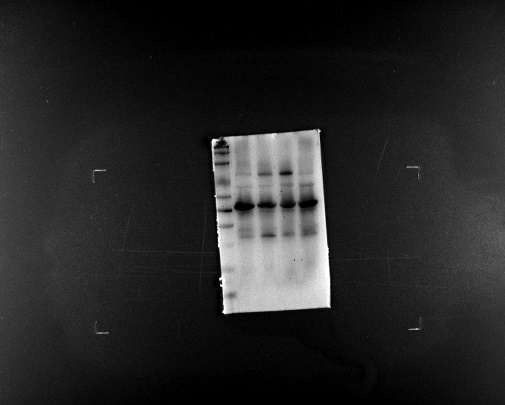


GPX4

β -actin

SLC7A111

ALOX15KD

20KD

40KD

50KD

70KD

100KD

***Cell***

ALOX15+β -actin Marker / control/CDDP/VE SLC7A11+β -actin Marker / control/CDDP/VE


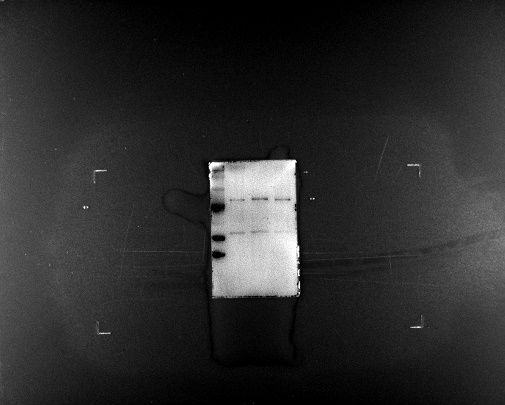

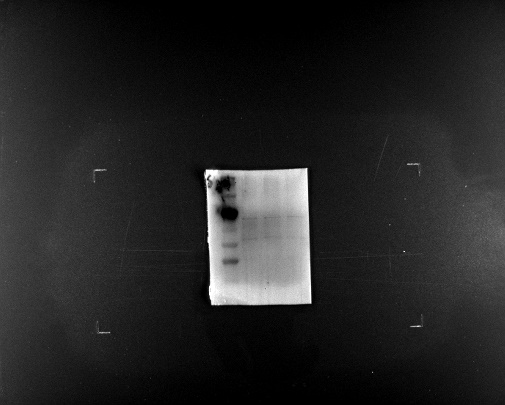


ALOX15 Marker /control//VE/CDDP/Marker/control//VE/CDDP/Marker/control//VE/CDDP/control//VE/CDDP β -actin


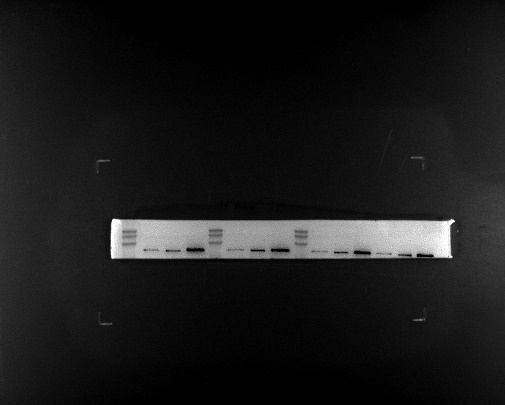

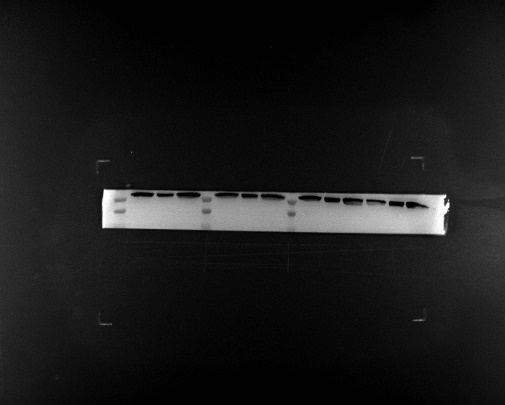


SLC7A11 control/CDDP/VE/Marker/ control/CDDP/Marker/ control/CDDP/VE/ Marker β -actin


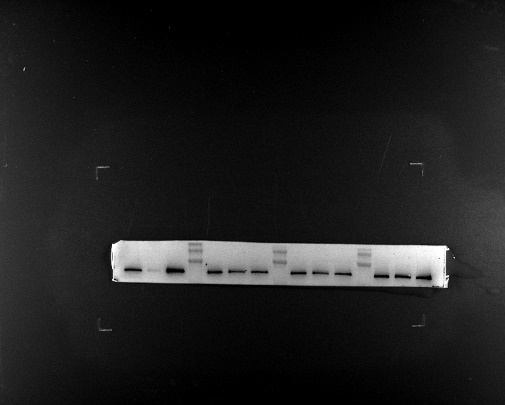

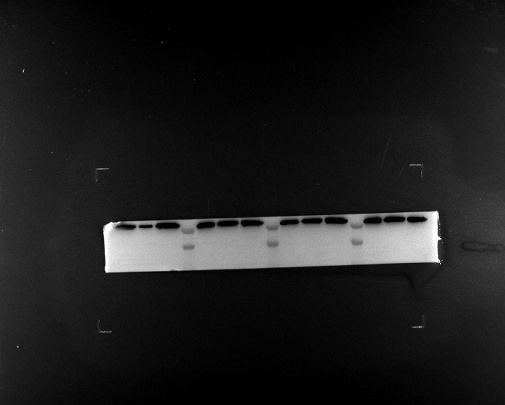


ACSL4 Marker /control/CDDP/VE/ control/CDDP/VE/ control/CDDP/VE β -actin


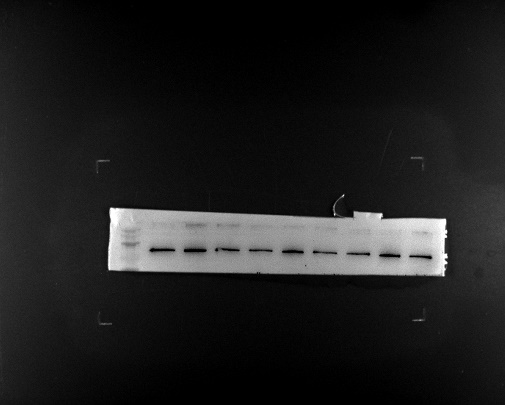

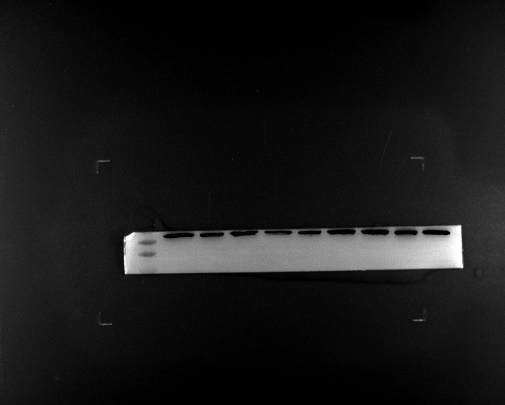


β -actin Marker / control/CDDP/VE


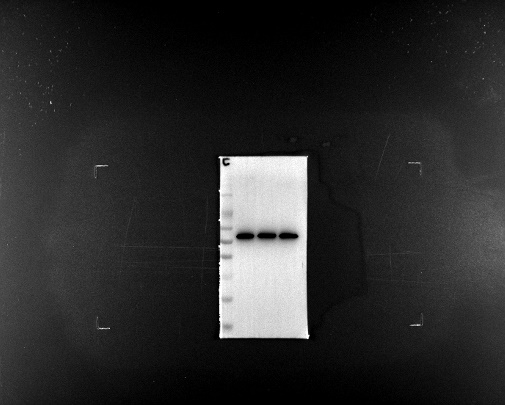


**WB image used for drawing in manuscript**

Ovarian tissue

ACSL4 Marker/control/CDDP/PBS/VE ALOX15 Marker/control/CDDP/PBS/VE


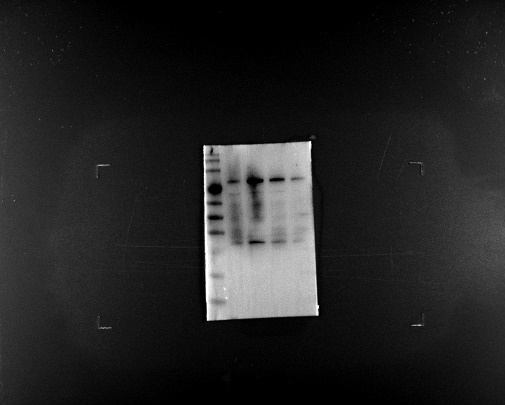

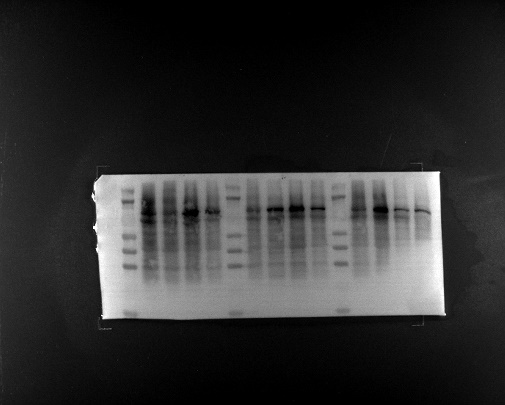


SLC7A11 Marker/control/CDDP/PBS/VE β -actin Marker/control/CDDP/PBS/VE


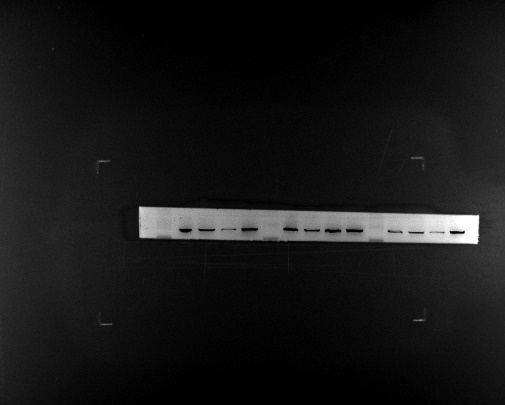

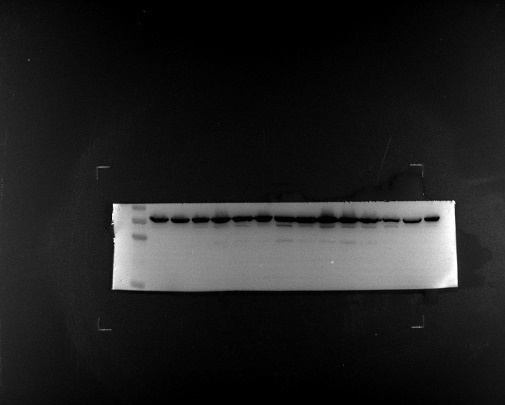


GPX4


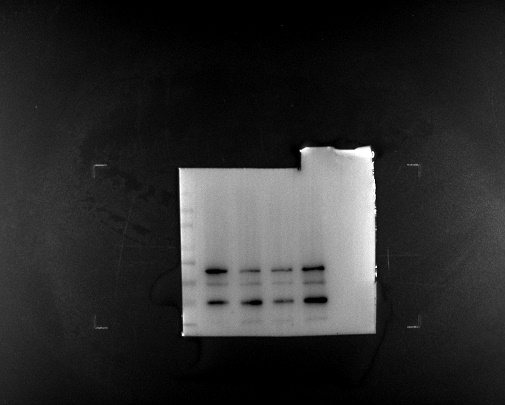


CELL

ACSL4 Marker/ control/CDDP/VE ALOX15 Marker / control/CDDP/VE


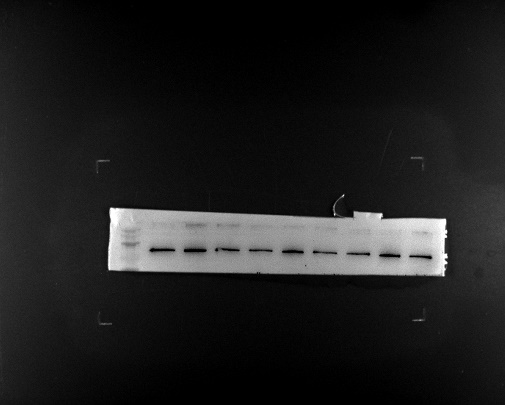

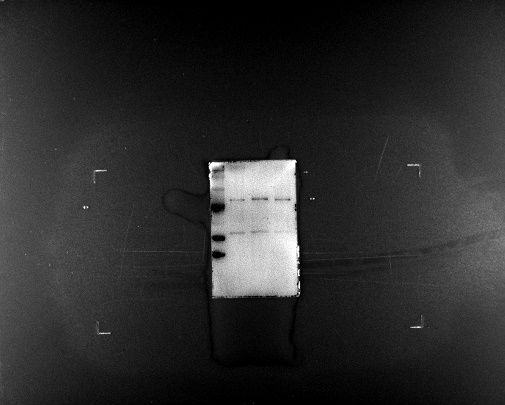


SLC7A11 Marker / control/CDDP/VE β -actin Marker / control/CDDP/VE


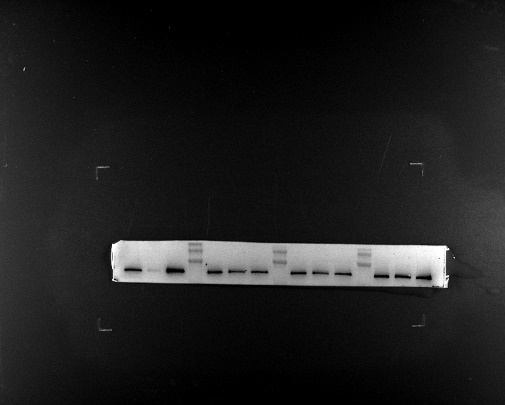

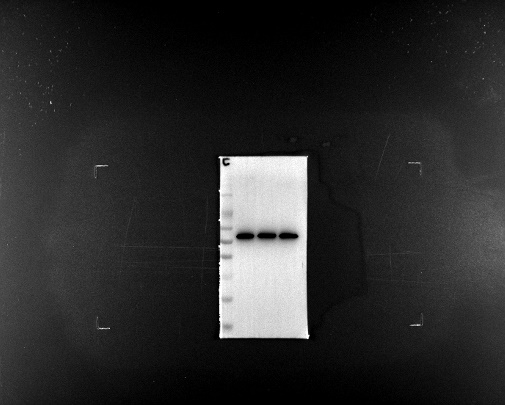

Supplement: Supplementary file 3 — Supplementary Information 3. [file 41598_2023_31712_MOESM3_ESM.docx]
